# Determination of Uranium Central-Field Covalency with 3*d*4*f* Resonant Inelastic X-ray Scattering

**DOI:** 10.1021/jacs.4c06869

**Published:** 2024-07-31

**Authors:** Timothy G. Burrow, Nathan M. Alcock, Myron S. Huzan, Maja A. Dunstan, John A. Seed, Blanka Detlefs, Pieter Glatzel, Myrtille O. J. Y. Hunault, Jesper Bendix, Kasper S. Pedersen, Michael L. Baker

**Affiliations:** † Department of Chemistry, The University of Manchester, Manchester, M13 9PL, U.K.; ‡ The University of Manchester at Harwell, Diamond Light Source, Harwell Campus, OX11 0DE, U.K.; ¶ Centre for Radiochemistry Research, The University of Manchester, Oxford Road, Manchester, M13 9PL, U.K.; § Department of Chemistry, Technical University of Denmark, 2800 Kongens Lyngby, Denmark; ∥ European Synchrotron Radiation Facility, 38000 Grenoble, France; ⊥ Synchrotron SOLEIL, LOrme des Merisiers, 91190 Saint-Auban, France; # Department of Chemistry, University of Copenhagen, 1172 Copenhagen, Denmark

## Abstract

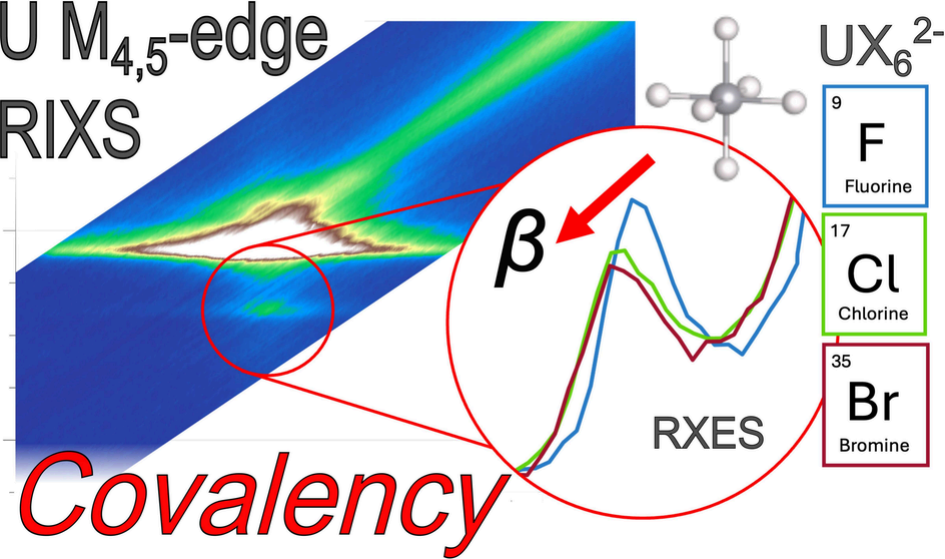

Understanding the nature of metal–ligand bonding is a major challenge in actinide chemistry. We present a new experimental strategy for addressing this challenge using actinide 3*d*4*f* resonant inelastic X-ray scattering (RIXS). Through a systematic study of uranium(IV) halide complexes, [UX_6_]^2–^, where X = F, Cl, or Br, we identify RIXS spectral satellites with relative energies and intensities that relate to the extent of uranium-ligand bond covalency. By analyzing the spectra in combination with ligand field density functional theory we find that the sensitivity of the satellites to the nature of metal–ligand bonding is due to the reduction of 5*f* interelectron repulsion and 4*f*-5*f* spin-exchange, caused by metal–ligand orbital mixing and the degree of 5*f* radial expansion, known as central-field covalency. Thus, this study furthers electronic structure quantification that can be obtained from 3*d*4*f* RIXS, demonstrating it as a technique for estimating actinide-ligand covalency.

## 1 Introduction

Covalency is a crucial concept for rationalizing physical and chemical properties and, as such, quantifying the covalent character in metal–ligand bonds is an essential challenge within inorganic chemistry.^1^ This challenge is particularly prominent for actinides due to their complex electronic structures and bonding interactions.^2−4^ Understanding the nature of actinide-ligand bonding is a central aim of actinide science, influencing chemical reactivity,^5−10^ redox potentials,^11−14^ catalysis,^15−17^ and magnetic properties.^18,19^

A covalent metal–ligand bond is formed by the mixing of frontier atomic orbitals to form a molecular orbital (MO) of both metal and ligand character. The influence of metal–ligand bonding on frontier MOs is mediated by two simultaneously acting phenomena: central-field covalency and symmetry-restricted covalency,^20^ expressed by
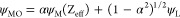
1Central-field covalency concerns the expansion of the metal-ion radial wave function (ψ_M_), which occurs as the effective cationic charge experienced by the valence shell (*Z*_*eff*_) is decreased by ligand electron donation. This expansion leads to a decrease in valence electron repulsion. Symmetry-restricted covalency originates from the mixing of ligand orbital (ψ_L_) character, given by the mixing coefficient α, into the MO (ψ_MO_). While central-field covalency is spherically symmetric, symmetry-restricted covalency depends on the point group symmetry of ψ_MO_. Symmetry-restricted covalent bonds, therefore, distort ψ_MO_ and delocalize ψ_M_, also leading to a reduction in valence electron repulsion.^20,21^

Experimentally, several techniques have been developed to deliver insight into actinide covalency, each with specific advantages. Electron paramagnetic resonance (EPR),^22^ nuclear magnetic resonance (NMR),^23−30^ and ligand K-edge absorption spectroscopy^31−36^ show sensitivity to symmetry-restricted covalency via *α*, providing vital contributions to the ongoing understanding of actinide-ligand bonding and covalency. However, these techniques also exhibit limitations that restrict broader applicability. NMR spectroscopy is generally limited to diamagnetic compounds, and EPR spectroscopy requires the compound of study to have an EPR active ground state with resolvable ligand super-hyperfine coupling; both techniques require ligand atoms to be nuclear spin active. Sensitivity to covalency in ligand K-edge spectroscopy requires well-resolved pre-edge features and has experimental challenges associated with the use of low energy X-rays for many common coordinating ligand elements (e.g., C, N, O, F), requiring ultrahigh vacuum conditions.

Cl ligand K-edge studies have provided a means to evaluate variation in symmetry-restricted covalency through the Group IV transition metals to U in [MCl_6_]^2–^, where M = Ti, Zr, Hf, and U.^32^ Analysis of these spectra reveals large pre-edge intensities due to *d* orbital *t*_2*g*_ covalency, which increases on progressing down the group from Ti through to Hf and a further increase in going to U. Furthermore, the Cl K pre-edge of [UCl_6_]^2–^ also shows lower energy transitions, providing clear evidence of 5*f* symmetry-restricted covalency.^32^ These insights and relativistic wave function-based simulations agree in the assignment of a small (relative to *d* orbital) but significant contribution of symmetry-restricted covalent mixing of Cl 3*p* with U 5*f* orbitals.^36^ Further studies at the Cl K-edge for early actinides in [MCl_6_]^2–^, where M = Th, U, Np, and Pu, all show evidence of similar amounts of 5*f* symmetry-restricted covalency.^34,36^

The study of electrostatic repulsion between pairs of electrons within a partially filled shell can also be used to probe metal–ligand bonding interactions. Schäffer and Jørgensen^37^ exploited this and showed that it is possible to organize ligand and central metal-ion by the relative reduction in interelectron repulsion (*F*^*k*^) on moving from a free-ion to an ion within a molecular complex, expressed in terms of the β parameter:
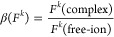
2They proposed to call this series the nephelauxetic (a neo-Greek word meaning cloud expanding) series, explaining the origin of the nephelauxetic effect as a combination of central-field and symmetry-restricted covalency. Contemporary transition metal photon-in photon-out X-ray spectroscopies^38,39^ exhibit sensitivity to the repulsion between valence *d*-electrons, from which the nephelauxetic effect can be quantified. For instance, transition metal Kβ emission spectroscopies are applied to probe covalency via sensitivity to 3*p*-3*d* or 3*p*-4*d* Slater integrals^40^ for intershell spin-exchange (*G*^*k*^).^41−43^ Similarly, transition metal 1*s*2*p* resonant inelastic X-ray scattering (RIXS) is a probe of the nephelauxetic effect via the analysis of splittings that relate directly to Slater integrals for interelectron repulsion (*F*^*k*^).^44,45^

**Figure 1:**
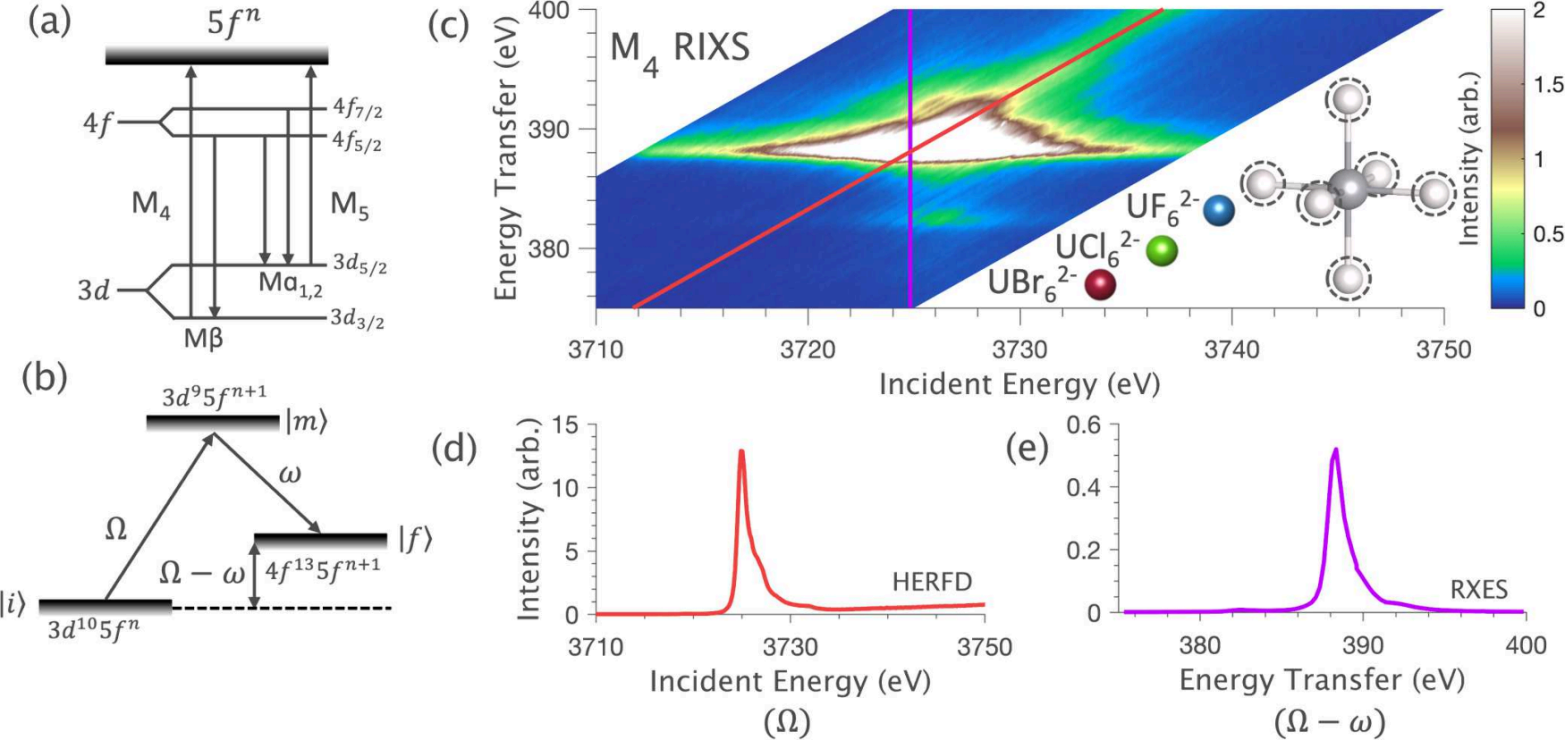
Schematics showing (a) the absorption and emission processes associated with actinide 3*d*4*f* RIXS, and (b) the relationship between incident (Ω), emitted (ω), and transfer photon energies (Ω–ω), and the RIXS initial |*i*⟩, intermediate |*m*⟩, and final |*f*⟩ state configurations. (c) An example 3*d*4*f* RIXS plane measured at the U M_4_-edge. (d) The diagonal red line indicates the HERFD cut (see text). (e) The vertical purple line indicates the RXES cut (see text). The inlay to (c) shows a molecular structure depicting the [UX_6_]^2–^ complexes studied in this work. The colors shown in the legend are used consistently throughout: blue ([UF_6_]^2–^), green ([UCl_6_]^2–^), and red ([UBr_6_]^2–^).

An increasingly large body of work shows that 3*d*4*f* RIXS exhibits sensitivity to actinide electronic structure and bonding.^46−56^ This two-photon process involves tuning the incident photon energy to a 3*d* → 5*f* absorption resonance and is followed by the detection of fluorescence photons from a 4*f* → 3*d* electronic relaxation. Due to spin–orbit splitting of the core levels, the absorption process involves a 3*d*_5/2_ (M_5_) and a 3*d*_3/2_ (M_4_) absorption edge, separated in energy by ca. 175 eV for U. The respective emission lines are denoted as Mα_1,2_ (4*f*_7/2_ and 4*f*_5/2_ → 3*d*_5/2_) and Mβ (4*f*_5/2_ → 3*d*_3/2_). These processes, illustrated in Figure 1a, are governed by electric dipole selection rules. The RIXS intensity is recorded as a function of both the incident photon energy (Ω) and the energy of the emitted photons (ω) and is represented on a two-dimensional plane as a function of Ω and the energy transfer between initial and final states (Ω – ω),^39^ shown in Figure 1b. Such a RIXS plane, at the M_4_ absorption edge and the Mβ emission line, is depicted in Figure 1c. The main 5*f* absorption features can be discerned by taking a constant emission energy cut diagonally through the RIXS plane (Figure 1d), known as HERFD (high energy resolution fluorescence detection). The fine structure in the emission profile can be resolved from a vertical cut through the RIXS plane at the resonant excitation energy (Figure 1e), known as RXES (resonant X-ray emission spectroscopy).^57^

In this work, we report 3*d*4*f* RIXS to carry a quantitative sensitivity to actinide metal–ligand bonding and covalency in a study of the structurally analogous U(IV) hexahalide series [UX_6_]^2–^, where X = F, Cl, and Br. We identify halide-dependent RIXS satellite intensities that carry sensitivity to metal–ligand bonding via the nephelauxetic effect. The observed RIXS satellites are presented to be sensitive to both central-field (via *Z*_*eff*_) and symmetry-restricted covalency (via *α*). The RXES component of the RIXS is found to carry the strongest sensitivity to bonding due to the nephelauxetic reduction of 4*f*-5*f* intershell spin-exchange interactions. The identified sensitivity to covalency is related to the measured RIXS spectra using ligand field density functional theory (LFDFT)^58^ from which the contributions of covalency, spin–orbit coupling, and ligand field splitting are included nonempirically. The LFDFT results are used to simulate the measured RIXS planes for direct comparison with the experimental data, providing valuable insight into the sensitivity of RIXS to actinide bonding and the importance of central-field covalency contributions to actinide-ligand bonds.

## 2 Results and Analysis

### 2.1 Experimental RIXS Data

**Figure 2:**
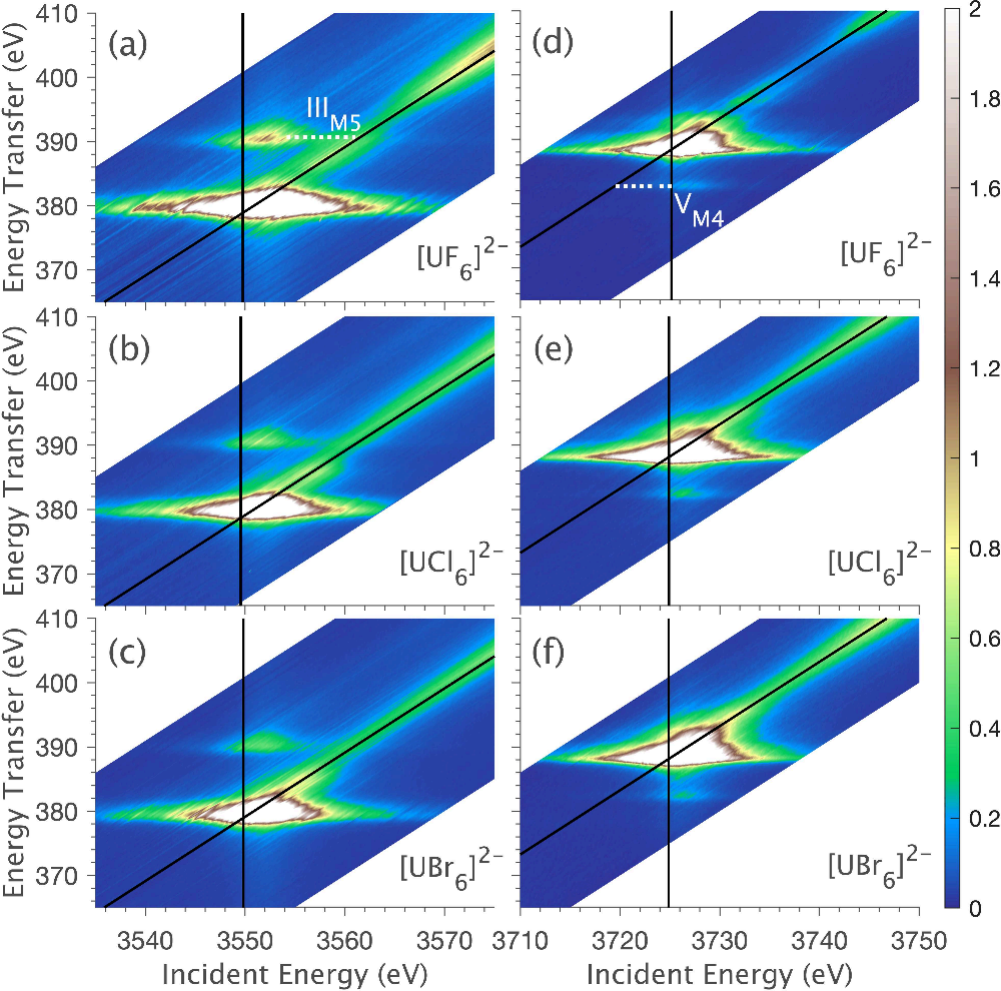
[UX_6_]^2–^ 3*d*4*f* RIXS planes measured at the M_5_-edge (a-c) and M_4_-edge (d-f). The diagonal black lines indicate the cuts taken to obtain HERFD spectra; the vertical black lines indicate RXES cuts. The white dashed lines show how the intensities from features III_M5_ and V_M4_ contribute to both the HERFD and the RXES cuts.

**Figure 3:**
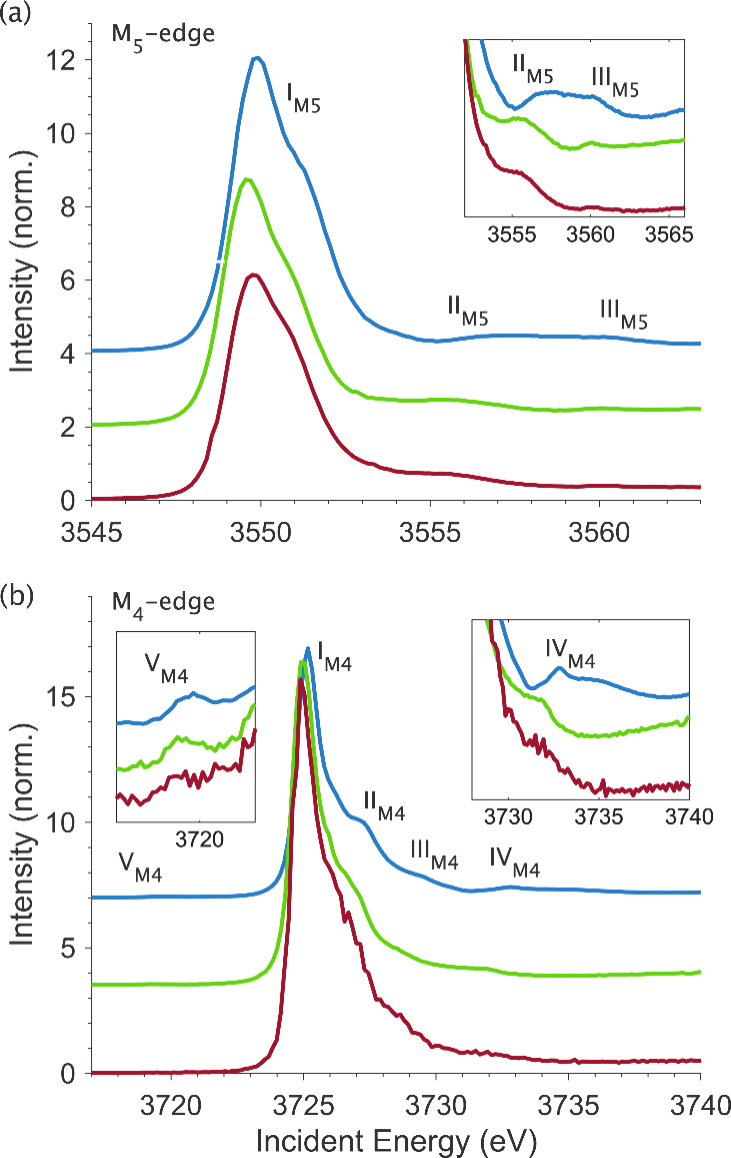
Experimental M_5_-edge (a) and M_4_-edge (b) HERFD spectra for [UF_6_]^2–^ (blue, *top*), [UCl_6_]^2–^ (green, *middle*), and [UBr_6_]^2–^ (red, *bottom*), with inset plots highlighting the identified satellite features.

The 3*d*4*f* RIXS planes obtained for [UF_6_]^2–^, [UCl_6_]^2–^, and [UBr_6_]^2–^ are shown in Figure 2. The M_5_-edge HERFD spectra in Figure 3a correspond to the diagonal cuts in the M_5_-edge RIXS planes taken at the maximum of the Mα_1_ (4*f*_7/2_ → 3*d*_5/2_) emission line (Figure 2a-c). The spectra are dominated by the resonant M_5_ absorption edge (3*d*_5/2_ → 5*f*), centered around 3550 eV incident energy, labeled I_M5_. Satellite features II_M5_ and III_M5_ are identified around 3555 and 3560 eV, respectively; their exact energy positions and intensity profiles are found to vary between the [UX_6_]^2–^ complexes (see Figure 3a inlay).

The M_4_-edge HERFD spectra, taken at the maximum of the Mβ (4*f*_5/2_ → 3*d*_3/2_) emission line, are shown in Figure 3b. The resonant M_4_ absorption edge (3*d*_3/2_ → 5*f*) feature is centered around 3725 eV incident energy, labeled I_M4_. There is a small variation in the energy position of I_M4_, with the maximum absorption energy of [UF_6_]^2–^ being slightly higher than that for [UCl_6_]^2–^ and [UCl_6_]^2–^ being slightly higher than that for [UBr_6_]^2–^.

High-energy shoulders II_M4_ and III_M4_ are resolved around 3727 and 3729 eV. A high energy satellite feature IV_M4_ is identified at around 3732 eV, and a weak pre-edge satellite V_M4_ is identified at around 3719 eV. The exact energy positions and intensity profiles of satellite features IV_M4_ and V_M4_ are halide-dependent.

**Figure 4:**
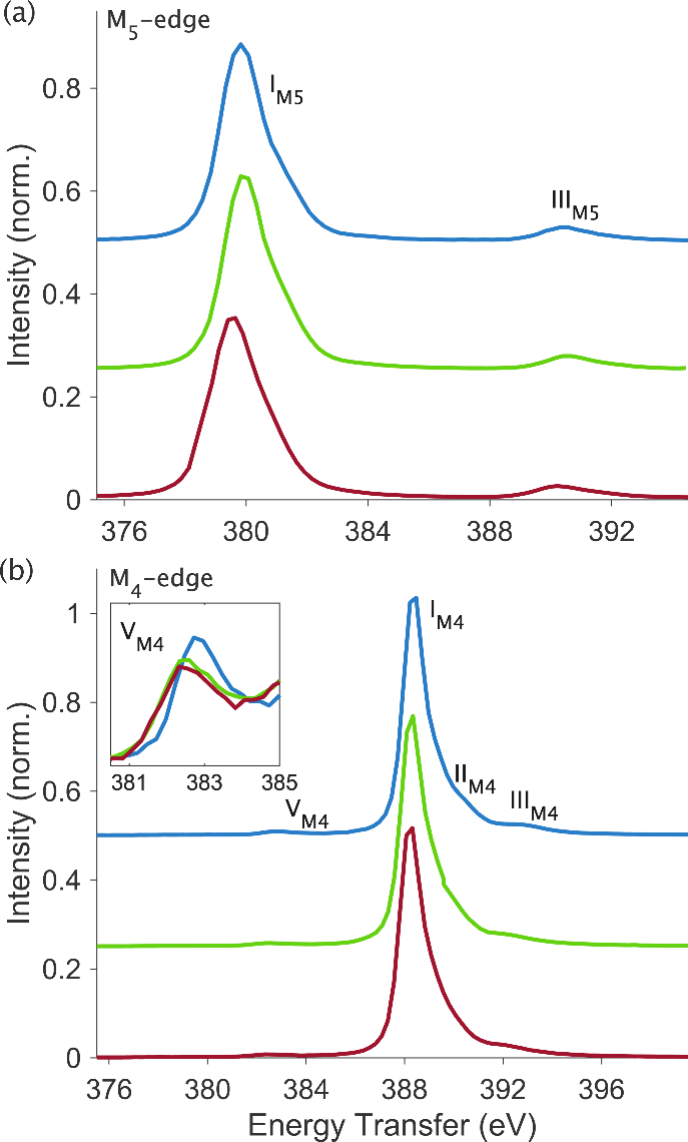
Experimental M_5_-edge (a) and M_4_-edge (b) RXES spectra for [UF_6_]^2–^ (blue, *top*), [UCl_6_]^2–^ (green, *middle*), and [UBr_6_]^2–^ (red, *bottom*). The M_5_-edge spectra show the Mα_1_ (I_M5_) and Mα_2_ (III_M5_) emission lines. The M_4_-edge spectra show the Mβ (I_M4_) emission line and additional features. The inlay shows an overlay of satellite feature V_M4_.

Figure 4a compares the RXES spectra at the M_5_-edge, corresponding to the vertical cuts through the RIXS planes. The spectra for the [UX_6_]^2–^ series are very similar and are dominated by two intense features that are centered at around 380 and 390 eV energy transfer, labeled I_M5_ and III_M5_. These correspond to the Mα_1_ (4*f*_7/2_ → 3*d*_5/2_) and Mα_2_ (4*f*_5/2_ → 3*d*_5/2_) emission lines, respectively, and their energy separation is defined by the spin–orbit coupling of the 4*f*^13^ part of the final state electron configuration.^50,59^ With reference to the RIXS plane (Figure 2), it is evident that features I_M5_ and III_M5_ in both the RXES and the HERFD directly correspond to the same RIXS feature. The broad profile in the incident energy dimension of feature III_M5_ is such that it extends to the area of the RIXS plane, which contributes to the HERFD intensity. Thus, we attribute satellite feature III_M5_ in the M_5_-edge HERFD to the residual intensity from the Mα_2_ emission line. This observation emphasizes the importance of the RIXS plane for the spectral interpretation of M_5_-edge HERFD spectra.

The M_4_-edge RXES spectra in Figure 4b are dominated by the Mβ (4*f*_5/2_ → 3*d*_3/2_) emission line, labeled I_M4_, around 388 eV energy transfer. Two high energy shoulders are resolved around 392 eV, labeled II_M4_ and III_M4_. A satellite feature is identified around 383 eV, labeled V_M4_. Features I_M4_, II_M4_, and III_M4_ correspond to the equivalent features identified in the HERFD and are associated with the main M_4_-edge (Mβ) RIXS process. The satellite feature V_M4_ is highlighted in the inlay plot of Figure 4b. Such a feature has been reported previously in 3*d*4*f* RIXS studies, however, no assignment of its origin has been made.^50^ The current data shows the energy position and intensity of V_M4_ to clearly vary between the [UX_6_]^2–^ complexes, with dependence on the identity of the bonding halide ligand. This implies that the feature carries sensitivity to the magnitude of 4*f*-5*f* spin-exchange interactions in the 4*f*^13^5*f*^3^ RIXS final state, which is perturbed by the bonding ligand environment. This assignment is supported by comparison with the M_4_-edge RXES of the hexavalent uranyl complex [UO_2_Cl_4_]^2–^ (Figure S1), for which no equivalent satellite feature is identified. The lack of feature in the U(VI) complex is attributed to a smaller spin moment in the 4*f*^13^ 5*f*^1^ RIXS final state configuration and large symmetry-restricted covalency contributions to bonding,^60^ both of which result in weaker 4*f*-5*f* spin-exchange interactions. Hence, we assign the RXES satellite V_M4_ to 4*f*-5*f* spin-exchange interactions. Figure 2 highlights how the broad profile of V_M4_ in the incident energy dimension leads to its contribution to the HERFD as a pre-edge intensity. We thus identify the origin of the weak pre-edge satellite V_M4_ in the M_4_-edge HERFD and expect it to also carry sensitivity to the final state 4*f*-5*f* spin-exchange interactions.

To explore the dependence of halide identity on the bonding nature of the [UX_6_]^2–^ complexes, we performed ground state electronic structure calculations, Section 2.2. In Section 2.3 we use ligand field density functional theory to parametrize multiplet simulations to model the 3*d*4*f* RIXS process and provide further insights into the assignments made above.

### 2.2 Ground State Electronic Structure Calculations

To investigate the ground state electronic structure of the [UX_6_]^2–^ complexes, Löwdin population analysis of DFT calculated molecular orbitals was performed. Calculations were performed assuming a high-spin 5*f*^2^ ground state configuration, and the resulting molecular orbitals of majority U 5*f* parentage were identified. The total 5*f* character of these orbitals shows evidence of some symmetry-restricted covalency, with U 5*f* -halide *p* mixing increasing on descending the halide group, leading to a small but significant decrease in U 5*f* character from [UF_6_]^2–^ (93.7%) to [UCl_6_]^2–^ (91.0%) to [UBr_6_]^2–^ (90.7%).

**Figure 5:**
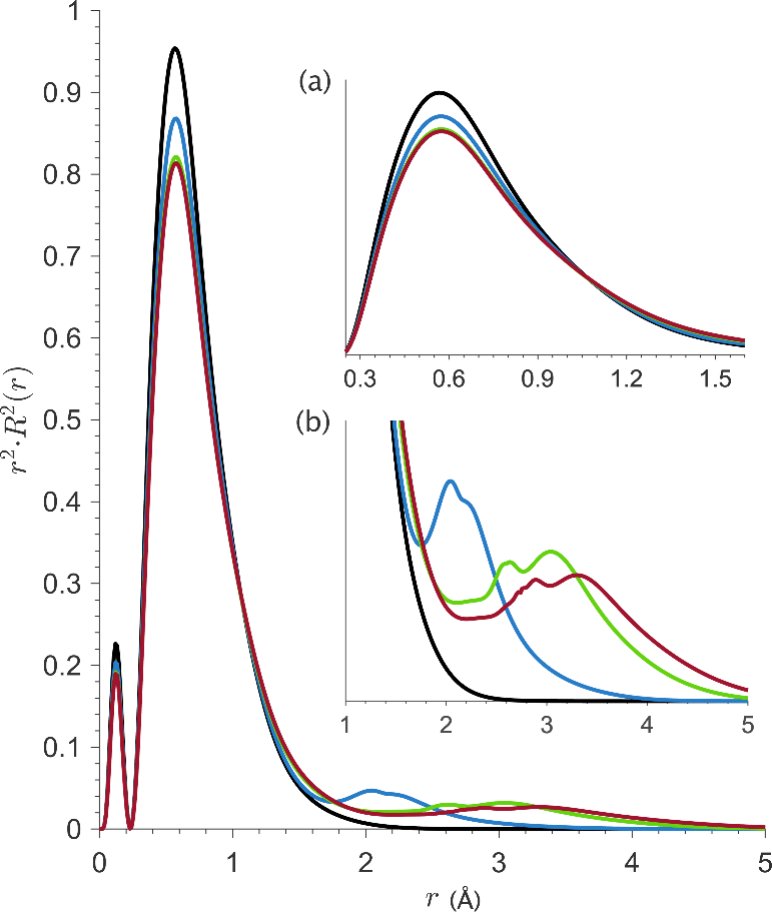
Radial distribution functions *r*^2^·*R*_MO_^2^(*r*) plotted for [UF_6_]^2–^ (blue), [UCl_6_]^2–^ (green), and [UBr_6_]^2–^ (red), where *R*_MO_ = |ψ_MO_|^2^. Also plotted is *r*^2^·*R*_5*f*_^2^(*r*) for the U(IV) free-ion (black), where *R*_5*f*_ = |ψ_5*f*_|^2^. Subplot (a) highlights the expansion of ψ_MO_ relative to ψ_5f_ in the U(IV) free-ion upon U–X bonding, while subplot (b) highlights the distortions to ψ_MO_ at *r* values close to the average U–X bond distances.

Figure 5 shows the radial distribution functions (RDFs) of these majority 5*f* molecular orbitals, *r*^2^·*R*_MO_^2^(*r*), plotted for each [UX_6_]^2–^ calculation. For comparative purposes, we also plot *r*^2^·*R*_5*f*_^2^(*r*) calculated for a U(IV) free-ion. The impact of metal–ligand bonding and the related influence of central-field and symmetry-restricted covalency can be interpreted with reference to these plots. In going from the U(IV) free-ion to the [UX_6_]^2–^ molecular complexes, an expansion of *r*^2^·*R*_MO_^2^(*r*) is observed. This central-field covalency effect infers a decrease in the effective cationic charge (*Z*_*eff*_) as ligand charge donation at the U 5*f* orbitals increases. The RDFs exhibit small distortions at *r* values close to the average U–X bond lengths (2.17 2.62, and 2.75 Å, for X = F, Cl, and Br, respectively). Noting their absence in *r*^2^·*R*_5*f*_^2^(*r*) for the U(IV) free-ion, these distortions arise from symmetry-restricted covalent interactions between the U 5*f* and halide valence orbitals, resulting in additional 5*f* orbital delocalization following the F < Cl < Br trend, in line with the Löwdin population analysis. Taken together, these phenomena infer a halide dependent nephelauxetic effect.

To predict the nephelauxetic effect quantitatively for the [UX_6_]^2–^ series, we turn to an *ab initio* ligand field theory (AILFT)^61−63^ analysis of state averaged (SA) complete active space self-consistent field (CASSCF) calculations at the *N*-electron valence perturbation theory (NEVPT2) level, using DFT orbitals described above as our initial starting point. From the AILFT, ligand field parameters are determined, including the Slater integrals describing 5*f* Coulomb repulsion (*F*_5*f*5*f*_^2^, *F*_5*f*5*f*_^4^, and *F*_5*f*5*f*_^6^), the relativistic spin–orbit coupling constant (ζ_5*f*_), and the 5*f* ligand field splitting. The reduction of *F*_5*f*5*f*_^*k*^ values for the [UX_6_]^2–^ complexes relative to the free-ion calculated value is expressed in terms of the nephelauxetic reduction factor (β) in Table 1.

**Table 1:** AILFT Derived Slater Integrals (*F*^*k*^) Describing 5*f*-5*f* Inter-electron Repulsion Expressed as a Fraction of the Value Calculated for a U(IV) Free-Ion (β)^a^

	β(*F*_5*f*5*f*_^2^)	β(*F*_5*f*5*f*_^4^)	β(*F*_5*f*5*f*_^6^)
[UF_6_]^2–^	0.95	0.91	0.92
[UCl_6_]^2–^	0.89	0.91	0.91
[UBr_6_]^2–^	0.88	0.91	0.90

a Absolute values are given in Table S1.

The trend in β aligns with the analysis of the populations and RDFs of the MOs of 5*f* parentage, with a reduction in β correlating with the extent of U 5*f* radial expansion and U 5*f* ligand *p* orbital mixing. The calculated values of β(*F*_5*f*5*f*_^2^) show the clearest trend, with the most significant reduction for [UBr_6_]^2–^, followed by that for [UCl_6_]^2–^, and the smallest reduction for [UF_6_]^2–^. The pronounced sensitivity in *F*_5*f*5*f*_^2^ stems from the radial contribution reaching its terminal value at a larger atomic radius (Figure S2).^21^ Consequently, Slater integrals of lower order (i.e., *F*_5*f*5*f*_^2^) can be expected to carry greater sensitivity to the external influence of ligand interactions than higher order integrals (i.e., *F*_5*f*5*f*_^4^, *F*_5*f*5*f*_^6^), *vide infra*.

**Figure 6:**
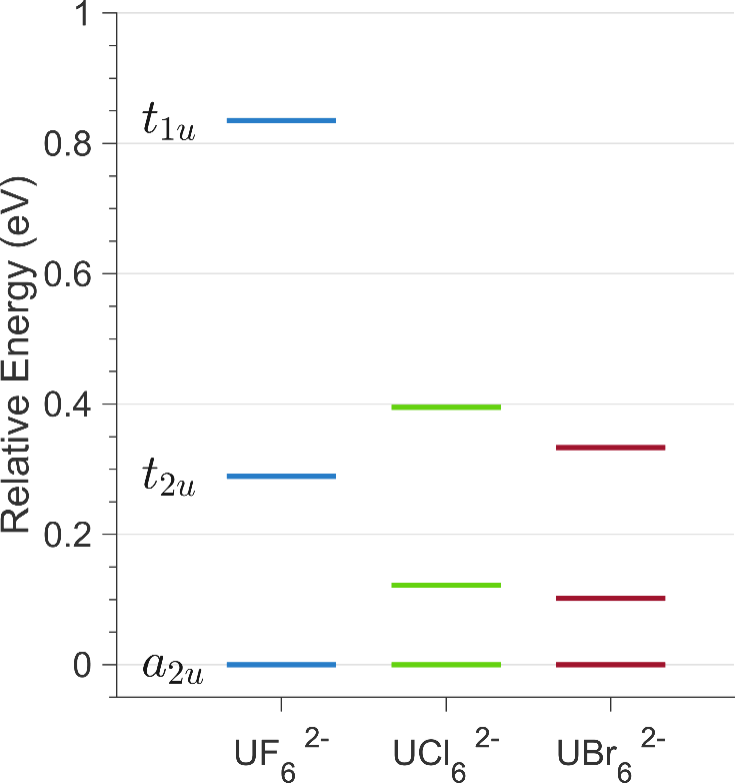
Relative 5*f* ligand field splittings calculated by AILFT are represented on an energy level diagram for each complex. The energies are given relative to the energy of the *a*_2*u*_ orbital, set to 0 eV. The values are given in Table S2.

Figure 6 shows the calculated 5*f* ligand field splittings for each complex. In *O*_*h*_ symmetry, the energies of the molecular orbitals of 5*f* parentage are reduced to three irreducible representations, *a*_2*u*_, *t*_2*u*_, and *t*_1*u*_, ordered from lowest to highest relative energy. The ligand field introduced by the F^–^ anion induces a much larger splitting of the 5*f* orbitals relative to that of the Cl^–^ or Br^–^ anions. These splittings are consistent with previous theoretical studies, and also correlate with reported experimental observations of *f*-*f* transitions to higher energies in [UF_6_]^2–^ compared to [UCl_6_]^2–^ and [UBr_6_]^2–^, following the spectrochemical series.^64−66^

### 2.3 Ligand Field Density Functional Theory Analysis of RIXS

For the inclusion of core-holes associated with the RIXS intermediate and final states we turn to the ligand field density functional theory (LFDFT) implemented in the Amsterdam Density Functional (ADF) code.^67,68^ The LFDFT aims to solve multiplet structure from first-principles, utilizing the average of configuration (AOC) DFT method to define an active space via the selective occupation of frozen Kohn–Sham molecular orbitals.^69^ This approach has been shown to accurately model the multiplet structure of 3*d*^10^5*f*^2^ → 3*d*^9^5*f*^3^ excitations in ThO_2_, UO_2_, and PuO_2_.^58,70^ We, therefore, extend this method to provide an *ab initio* set of ligand field parameters describing the [UX_6_]^2–^ systems in each of the ground (5*f*^2^), intermediate (3*d*^9^5*f*^3^), and final (4*f*^13^5*f*^3^) configurations of the RIXS process.

**Figure 7:**
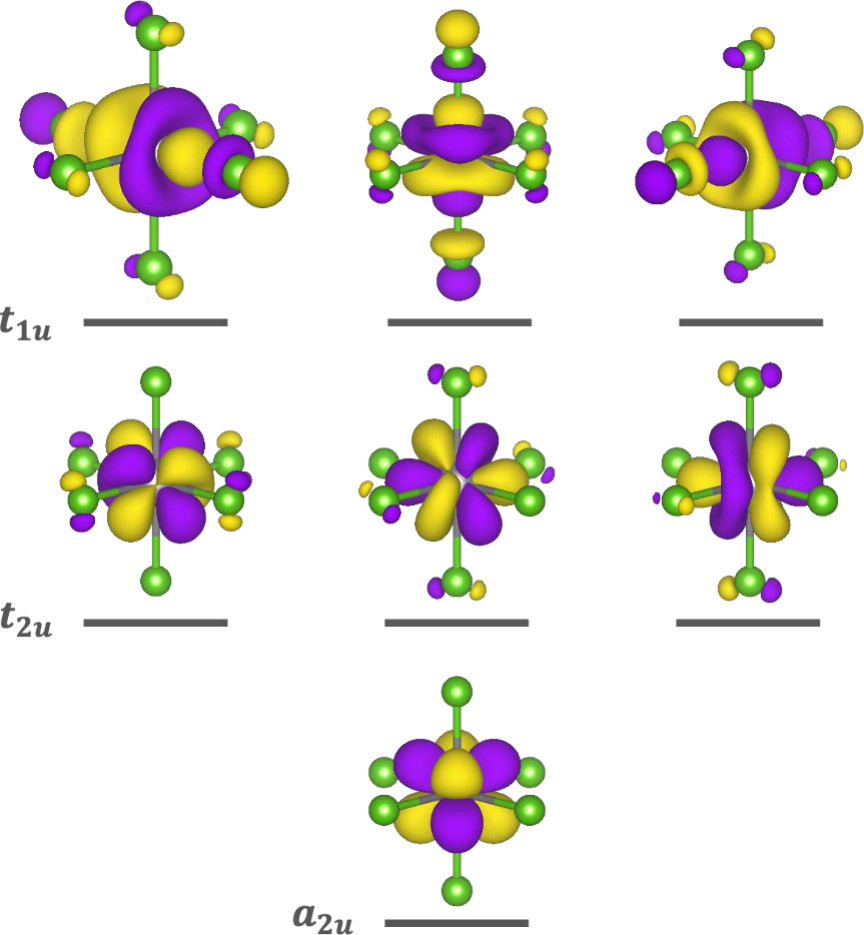
Representation of the energy splitting of the 5*f* Kohn–Sham orbitals of [UCl_6_]^2–^, obtained from a ground state AOC calculation. The symmetry labels refer to the irreducible representations of the 5*f* orbitals in the *O*_*h*_ point group.

The Kohn–Sham MOs with the most significant U 5*f* character calculated for the [UCl_6_]^2–^ ground state are shown in Figure 7; the equivalent MOs for [UF_6_]^2–^ and [UBr_6_]^2–^ are shown in Figure S4, as well as those for the intermediate and final states.

The MOs from the AOC calculations were used in the LFDFT analysis to obtain the Coulomb (*F*^*k*^) and exchange (*G*^*k*^) Slater integrals, the relativistic spin–orbit coupling constants (ζ_*nl*_), and the 5*f* ligand field splittings in each of the ground, intermediate, and final RIXS configurations (Tables S3–S8).

**Figure 8:**
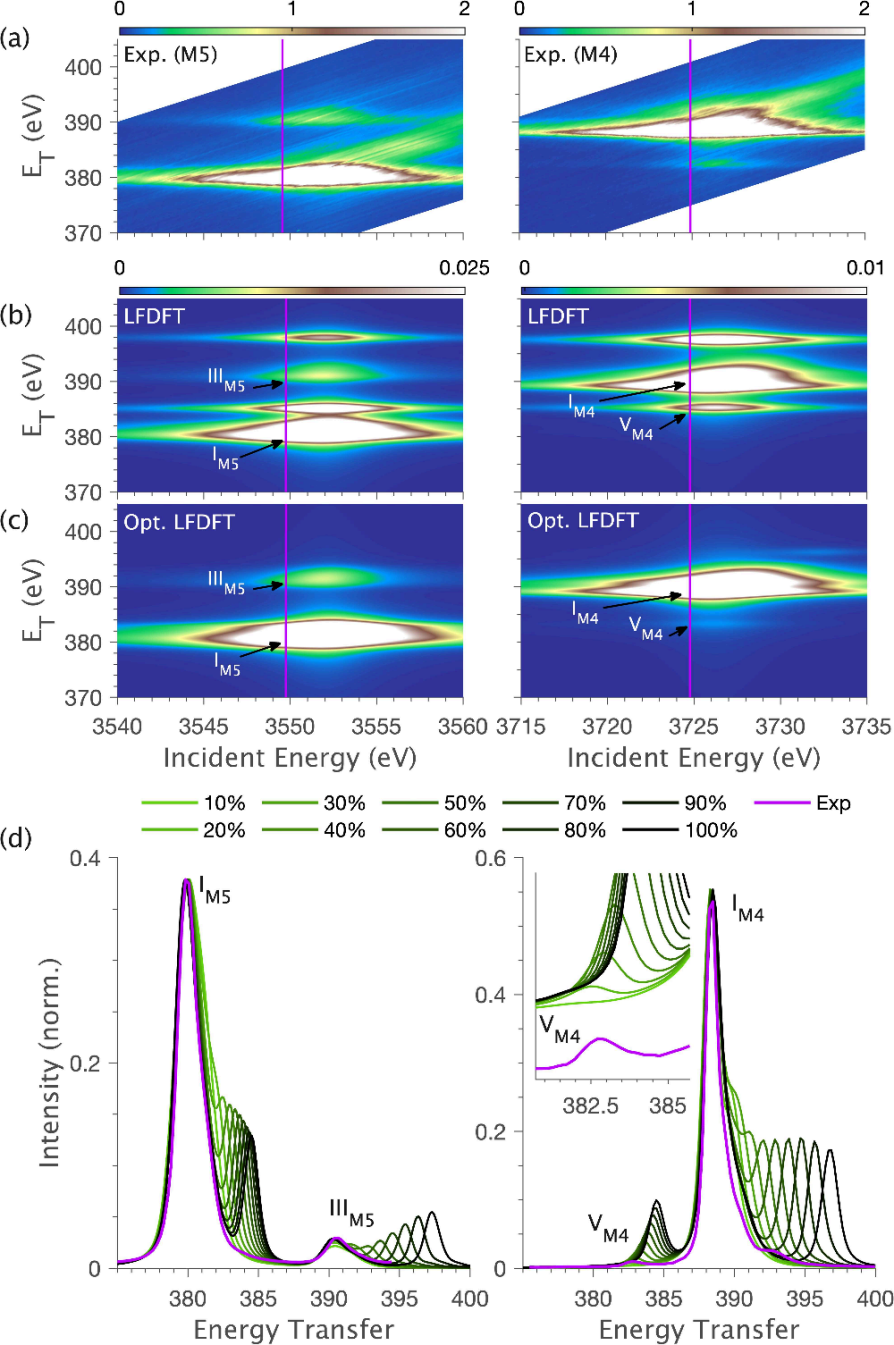
Experimental and simulated RIXS data for [UCl_6_]^2–^ at the M_5_ (left) and M_4_ (right) edges: (a) Experimental RIXS planes; (b) RIXS planes simulated *ab initio* using LFDFT parameters (Tables S3–S8); (c) optimized RIXS planes simulated with a 20.5% reduction of the LFDFT calculated 4*f*-5*f* Slater integrals to provide the best fit to experiment (optimized values given in Tables S9–S11); (d) RXES cuts showing the stepwise effect of decreasing the magnitude of 4*f*-5*f* Slater integrals.

The LFDFT results were then used to simulate RIXS planes *ab initio* with the ligand field multiplet theory. The result for [UCl_6_]^2–^ is shown in Figure 8b. The equivalent LFDFT RIXS planes for [UBr_6_]^2–^ and [UF_6_]^2–^ are similar (Figure S5). The LFDFT simulations of the [UX_6_]^2–^ series deviate, most notably in the RXES region of the RIXS planes. The experimental M_4,5_-edge RIXS planes for [UCl_6_]^2–^ are plotted alongside the equivalent LFDFT simulation in Figure 8a. The M_5_-edge LFDFT simulation reproduces the experimentally observed Mα_1_ (I_M5_) and Mα_2_ emission lines (III_M5_), with their relative energy splitting defined by the spin–orbit coupling of the 4*f* core-hole. However, the simulation also shows two additional features at energy transfers of ∼385 and 398 eV, which are not observed in the experimental data. The M_4_-edge LFDFT RIXS simulations reproduce the general line shape of the Mβ emission line (I_M5_), however, the low energy transfer satellite feature (V_M5_) is much more intense than experimentally observed. The simulation also includes a high-energy-transfer satellite (∼398 eV) that is not present in the experimental RIXS.

To determine the origin of the discrepancy in LFDFT RIXS simulations, specific electronic structure parameters were systematically varied away from the values given by LFDFT. These calculations reveal the observed chemical sensitivity of U(IV) 3*d*4*f* RIXS, showing that spectra are dominated by multiplet effects, with the spectral shape most strongly dependent on the 4*f*-5*f* intershell spin-exchange. In contrast, the spectra show only a minor sensitivity to the symmetry and magnitude of 5*f* ligand field splitting and 5*f* spin–orbit coupling (Figures S7–S8).

RXES cuts through the RIXS plane (Figure 8d) show most clearly how the reduction of 4*f*-5*f* Slater integrals (*G*_4*f*5*f*_^*k*^ and *F*_4*f*5*f*_^*k*^) directly affects the spectral shape. Reducing the 4*f*-5*f* Slater integrals to ∼20% of the LFDFT-derived values results in a greatly improved fit of the RXES. The *G*_4*f*5*f*_^0^ Slater integral has the strongest influence, with the best fit for [UCl_6_]^2–^ corresponding to a β(*G*_4*f*5*f*_^0^) = 0.17. The M_4_-edge RXES provides the most precise handle on β(*G*_4*f*5*f*_^0^), defined by the energy and intensity of the low energy transfer satellite V_M4_, whereas the M_5_-edge RXES can be reproduced with β(*G*_4*f*5*f*_^0^) values lower than 0.5.

**Figure 9:**
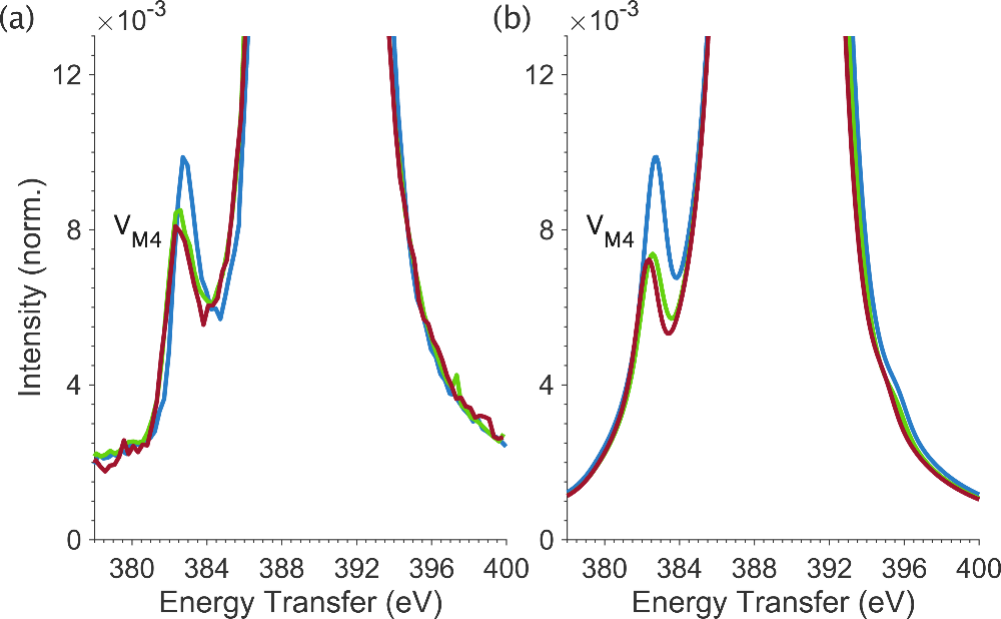
(a) Experimental RXES spectra for [UF_6_]^2–^ (blue), [UCl_6_]^2–^ (green), and [UBr_6_]^2–^ (red). (b) Equivalent RXES cuts through the simulated RIXS planes.

Optimized simulations of the [UCl_6_]^2–^ RIXS planes with scaled 4*f*-5*f* Slater integrals are shown in Figure 8c (Figure S6 for [UF_6_]^2–^ and [UBr_6_]^2–^). Comparison of experimental M_4_-edge RXES for the [UX_6_]^2–^ series is shown in Figure 9, with a focus on the low energy transfer satellite V_M4_, along with the optimized LFDFT simulations. The simulations accurately reproduce feature V_M4_, including differences in the energy and intensity of V_M4_ for [UF_6_]^2–^ relative to those of [UCl_6_]^2–^ and [UBr_6_]^2–^. Accurate reproduction of the observed differences in the energy and intensity of feature V_M4_ for [UCl_6_]^2–^ and [UBr_6_]^2–^ versus [UF_6_]^2–^ requires significant β(*G*_4*f*5*f*_^*k*^) nephelauxetic reduction factors of 0.20 for [UF_6_]^2–^ and 0.17 for [UCl_6_]^2–^ and [UBr_6_]^2–^. It is therefore shown that the Mβ RXES component of 3*d*4*f* RIXS is a sensitive probe of 5*f* metal–ligand bonding via the nephelauxetic effect, similar to Kβ XES that carries the same sensitivity for transition metals.^41−43^

The RIXS simulations including the scaled 4*f*-5*f* Slater integral values reproduce the M_5_-edge HERFD spectra (Figure S9). The simulations confirm that feature III_M5_ results from residual intensity from the Mα_2_ emission line. Satellite feature II_M5_ is identified as not originating from multiplet effects. Therefore, it is assigned to be a ligand-to-metal charge transfer satellite, most likely originating from *t*_1*u*_ symmetry charge donation.

**Figure 10:**
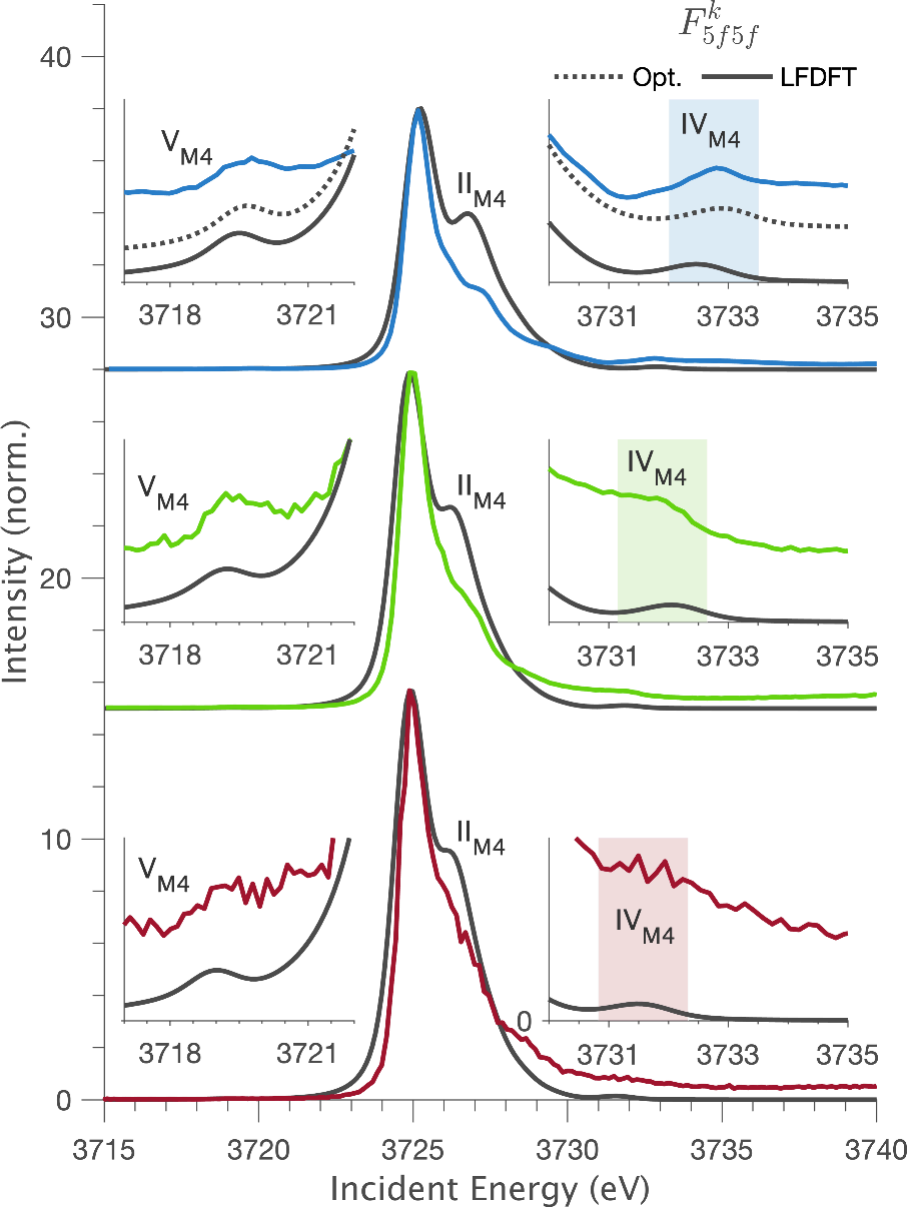
Experimental M_4_-edge HERFD cuts versus the optimized LFDFT simulations for [UF_6_]^2–^ (*top*), [UCl_6_]^2–^ (*middle*), and [UBr_6_]^2–^ (*bottom*). For [UF_6_]^2–^, slight tuning of *F*_5*f*5*f*_^*k*^ is required to accurately reproduce feature IV_M4_, whereas [UCl_6_]^2–^ and [UBr_6_]^2–^ are reproduced using 100% LFDFT *F*_5*f*5*f*_^*k*^ values. The optimized parameters are given in Tables S9–S11.

The M_4_-edge HERFD spectra exhibit more fine structure than the M_5_-edge. LFDFT simulations incorporating the reduced 4*f*-5*f* Slater integrals reproduce the M_4_-edge HERFD spectra, including the pre-edge feature V_M4_ and high energy satellite IV_M4_. We find satellite feature IV_M4_ carries a strong sensitivity to the *F*_5*f*5*f*_^2^ Slater integral which describes the degree of 5*f* Coulomb repulsion. Figure 10 shows that IV_M4_ is accurately predicted by LFDFT for [UCl_6_]^2–^ and [UBr_6_]^2–^. For [UF_6_]^2–^ a minor improvement can be obtained with a slight increase in *F*_5*f*5*f*_^2^. The HERFD analysis gives experimentally quantified β(*F*_5*f*5*f*_^2^) values of 0.84, 0.77, and 0.71 for the 4*f*^13^5*f*^3^ RIXS final state for [UF_6_]^2–^, [UCl_6_]^2–^, and [UBr_6_]^2–^, respectively. The trend in β(*F*_5*f*5*f*_^2^) follows the same trend obtained in the ground state by AILFT (Table 1) and correlates with the MO population analysis, which indicates increased 5*f* delocalization in going from [UF_6_]^2–^ to [UCl_6_]^2–^ and to [UBr_6_]^2–^. We therefore show that the U(IV) M_4_-edge HERFD is a probe of metal–ligand bonding via the nephelauxetic reduction of *F*_5*f*5*f*_^*k*^ Slater integrals describing 5*f*-5*f* electron interactions in the bonding molecular orbitals.

The simulations in Figure 10 show an overestimation in the intensity of shoulder feature II_M4_ relative to the experimental data. A survey of ligand field multiplet parameters finds that feature II_M4_ is not uniquely sensitive to one particular set of electronic structure parameters and also carries further sensitivity to the specific line shape broadening implemented in the RIXS simulation. Since our objective is to accurately simulate the full experimental RIXS data with as few free parameters as possible, we did not attempt to optimize the fit of II_M4_ any further.

## 3 Discussion

In recent years, M_4,5_-edge HERFD has matured into an essential method for determining actinide electronic structure, bonding, and speciation.^52,55,71^ An increasing number of studies demonstrate that the method carries sensitivity to oxidation state, 5*f* ligand field splitting, and covalency.^46−51,53,54,72−75^ It has also been shown that additional insights can be obtained by extending HERFD measurements to include 3*d*4*f* RIXS planes, particularly in identified variations in resonant versus nonresonant XES maxima.^49,50,73^ The present study reports the sensitivity of 3*d*4*f* RIXS to U(IV) metal–ligand bonding at both the M_5_-edge and the M_4_-edge. It is identified that the RXES part of the RIXS planes contains fine structures that are not captured via HERFD measurements. M_4_-edge RIXS is found to carry greater sensitivity to U(IV) bonding than the M_5_-edge RIXS. The M_4_-edge RXES cut through the RIXS reveals satellites with relative intensities and energies that depend on U(IV) bonding. Similarly, additional satellites with a U(IV) bonding sensitivity are identified in the M_4_-edge HERFD at incident energies between 5 and 8 eV above the absorption edge. The identified satellites highlight the importance of measuring RXES spectra in addition to HERFD spectra that extend to incident photon energies well above the absorption edge.

**Figure 11:**
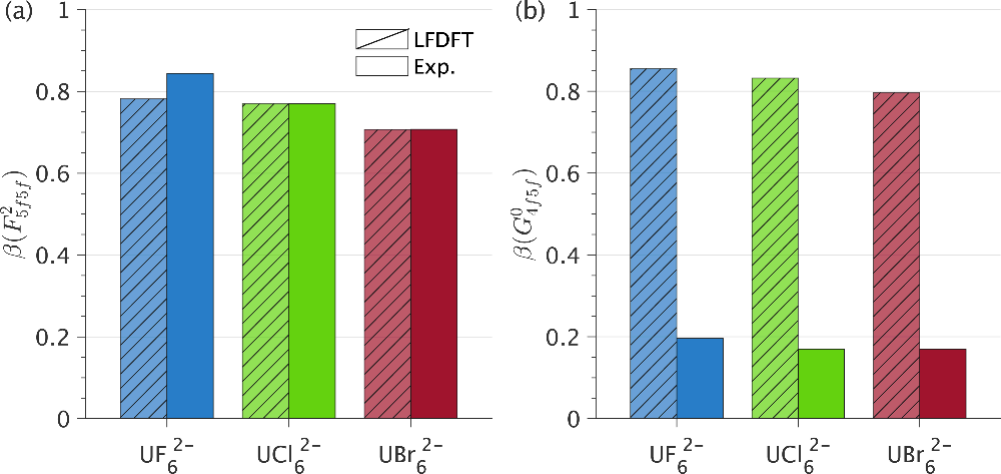
Nephelauxetic reduction parameters (β) for *F*_5*f*5*f*_^2^ (a) and *G*_4*f*5*f*_^0^ (b) determined from LFDFT (hatched bars) and from fitting to the 3*d*4*f* RIXS experiment (solid bars).

The relative energy and intensity of the identified M_4_-edge RIXS spectral satellites are analyzed to quantify the strength of 4*f*-5*f* intershell spin-exchange and 5*f*-5*f* Coulomb repulsion in the RIXS final state, defined by *G*_4*f*5*f*_^*k*^ and *F*_5*f*5*f*_^*k*^ Slater integrals. LFDFT calculations provide *ab initio* insight into the relationship between the observed spectral satellites and metal–ligand bonding interactions and the impact this has on Slater integrals. The calculations confirm the physical origin of the identified spectral satellites, demonstrating that [UX_6_]^2–^ RIXS spectra carry the greatest sensitivity to *F*_5*f*5*f*_^2^ and *G*_4*f*5*f*_^0^. The RIXS determined [UX_6_]^2–^ Slater integrals are significantly reduced relative to the U(IV) free-ion. This is most clearly expressed via the nephelauxetic reduction factor (β), the ratio between the Slater integral for a given complex relative to its free-ion Slater integral value (*c.f.*eq 2). Figure 11a shows the trends in β(*F*_5*f*5*f*_^2^) for [UF_6_]^2–^, [UCl_6_]^2–^, and [UBr_6_]^2–^ calculated by LFDFT versus the optimized best fits from the experimental data. β(*F*_5*f*5*f*_^2^) decreases in going from [UF_6_]^2–^ to [UBr_6_]^2–^, with good agreement between the experimental and LFDFT data with a significant reduction for all three complexes. For comparison, β(*F*^2^_3*d*3*d*_) values of 0.91, 0.86, and 0.85 are reported for the transition metal halide series [MnX_4_]^2–^ (X = F, Cl, Br, respectively) from 3*d*-3*d* spin-flip transitions in their electronic absorption spectra.^20^ These trends, and many others, show a systematic reduction from F to Cl to Br in accordance with the predicted halide nephelauxetic series.^76−86^ As introduced in Section 1, the nephelauxetic effect originates from a combination of central-field and symmetry-restricted covalency.^20^ For [UX_6_]^2–^, this assertion can be rationalized by analyzing DFT radial distribution functions (*cf.*Section 2.2). However, central-field and symmetry-restricted covalency contributions are inherently interlinked, and the deconvolution of their independent contributions cannot be experimentally partitioned. Despite this, the symmetry-restricted covalency in [UCl_6_]^2–^ can be estimated using Cl K-edge X-ray absorption spectroscopy (XAS), with a small contribution of ∼5.6% Cl 3*p* character mixed into U 5*f*(*t*_1*u*_,*t*_2*u*_) being reported.^32,34^ Therefore, to explain the strong reductions in β(*F*_5*f*5*f*_^2^) identified for [UX_6_]^2–^ a sizable central-field covalency contribution to the β(*F*_5*f*5*f*_^2^) nephelauxetic effect is deduced.

**Figure 12:**
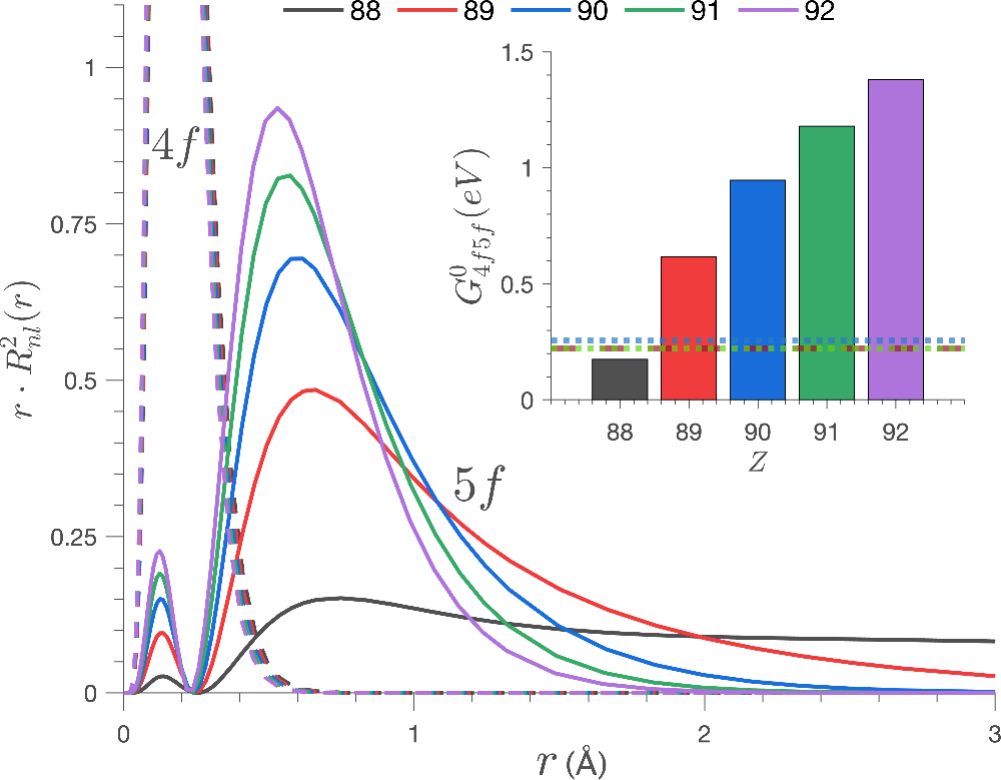
Calculated 4*f* and 5*f* RDFs plotted as a function of the nuclear charge (*Z*). (Inlay) Calculated radial integrals for *G*_4*f*5*f*_^0^ plotted as a function of nuclear charge, with experimentally determined values for [UF_6_]^2–^, [UCl_6_]^2–^, and [UBr_6_]^2–^ shown with horizontal dotted lines.

Figure 11b shows the trends in β(*G*_4*f*5*f*_^0^) for [UF_6_]^2–^, [UCl_6_]^2–^, and [UBr_6_]^2–^ calculated by LFDFT versus the optimized best fits from the experimental data. A decrease from [UF_6_]^2–^ to [UCl_6_]^2–^ to [UBr_6_]^2–^ is observed. However, the total reduction for *G*_4*f*5*f*_^0^ is much more pronounced than for *F*_5*f*5*f*_^2^, and while *F*_5*f*5*f*_^2^ is accurately predicted by LFDFT, the theoretical values obtained for *G*_4*f*5*f*_^0^ are approximately four times larger than those obtained by experiment. The *G*_4*f*5*f*_^0^ Slater integral is inherently more complex than *F*_5*f*5*f*_^2^ since it depends on the precise overlap and the relative phase of the 4*f* and 5*f* radial wave functions. Figure 12 shows how *G*_4*f*5*f*_^0^ is strongly affected by the reduction of the nuclear charge (*Z*). The figure shows the effect of varying *Z* on a U(IV) ion (from *Z* = 92 to *Z* = 88) as a means to modify the effective charge at the 4*f* and 5*f* shells, and the impact this has on the magnitude of *G*_4*f*5*f*_^0^. The 5*f* atomic wave function expands as *Z*_*eff*_ is decreased, whereas the influence on the 4*f* atomic wave function is much more subtle. The calculated influence of relativistic effects on 4*f* and 5*f* atomic orbitals for U(IV) are shown in Figure S3. These demonstrative calculations show how the 5*f* radial distribution is much more strongly influenced by relativistic effects than is 4*f*, and *G*_4*f*5*f*_^0^ is more heavily affected by relativistic effects than *F*_5*f*5*f*_^2^. Consequently, the 4*f* subshell acts as a benchmark for quantifying 5*f* radial expansion, making *G*_4*f*5*f*_^0^ a particularly sensitive probe of the 5*f* relativistic expansion and covalency in the 4*f*^13^5*f*^3^ RIXS final state.

From a computational perspective, the present study confirms that accurate simulation of M_4,5_-edge HERFD requires evaluating the full RIXS process described by the Kramers–Heisenberg formula.^87^ It is also identified that metal–ligand bonding contributions to Slater integrals are significant, and that predictions of Slater integrals via atomic Hartree–Fock theory, scaled to 80%,^87,88^ are inaccurate. It is shown that 5*f*-5*f* Coulomb repulsion can be accurately predicted by LFDFT, but 4*f*-5*f* spin-exchange cannot. Consequently, accurate simulation of HERFD data benefits from related RXES measurements to provide an experimental handle on spectral contributions originating from the presence of the 4*f* core-hole. Given known incompleteness errors originating in inner shells using ZORA,^89^ it will be informative to identify if improved quantification of spin-exchange can be obtained with alternatives.

In comparison with other experimental methods, the identified sensitivities of 3*d*4*f* RIXS to bonding indicate that the method exhibits several advantages for evaluating covalency and testing theoretical descriptions of actinide electronic structure. For instance, 3*d*4*f* RIXS does not rely on the actinide’s paramagnetic state or the ligand’s nuclear spin, and in contrast to ligand K-edge XAS, it is not reliant on absolute transition intensity standards since only relative spectral intensities are required. We are now expanding this method to a range of U oxidation states and other actinides. Based on this ongoing work, we predict the quantification of nephelauxetic effects by 3*d*4*f* RIXS and, in particular, RXES, to be universal to actinide element identity, ligand element identity, actinide oxidation state, and actinide coordination symmetry, making it particularly advantageous over existing techniques for the study of low symmetry systems, as well as systems consisting of bulky ligand environments. Furthermore, the technique is not limited to the study of molecular systems and can equivalently be applied to extended solids, amorphous samples, and dopants. Consequently, the method is expected to have the capacity to contribute widely to actinide analytical chemistry and material characterization.

## 4 Conclusions

The electronic structures of [UX_6_]^2–^, where X = F, Cl, and Br, have been rigorously investigated with 3*d*4*f* RIXS at both the M_4_ and M_5_ absorption edges. Previously unassigned spectral satellites have been identified and found to exhibit sensitivity to the nature of uranium-halide bonding interactions. 3*d*4*f* RIXS is presented to be a new method for estimating the nature of actinide-ligand covalency, complementary to methods such as NMR spectroscopy, pulsed-EPR spectroscopy, and ligand K-edge XAS. Sensitivity to bonding in 3*d*4*f* RIXS originates from variations in 5*f*-5*f* Coulomb repulsion and 4*f*-5*f* spin-exchange, defined by *F*_5*f*5*f*_^*k*^ and *G*_4*f*5*f*_^*k*^ Slater integrals. The RIXS spectra display the greatest sensitivity to *F*_5*f*5*f*_^2^ and *G*_4*f*5*f*_^0^ Slater integrals, which both exhibit strong reduction, with [UBr_6_]^2–^ and [UCl_6_]^2–^ having a more significant reduction than [UF_6_]^2–^. The identified sensitivity to bonding is interpreted with ligand field DFT to originate from symmetry-restricted metal–ligand covalency and 5*f* radial expansion (central-field covalency). Since 3*d*4*f* RIXS can be applied to actinide compounds with any ligand atom identity, nuclear spin, actinide oxidation state, or coordination symmetry, the reported methodology for estimating actinide-ligand bond covalency is expected to be widely applicable in actinide analytical chemistry and material characterization.

## 5 Methods

### 5.1 Experimental Section

#### Sample Preparation

***Caution!***^238^U isotopes are low specific-activity α-particle emitting radionuclides, and their use presents hazards to human health. This research was conducted in a radiological facility with appropriate analyses of these hazards and implementation of controls for the safe handling and manipulation of these toxic and radioactive materials. (NEt_4_)_2_[UF_6_]·7H_2_O (Et = ethyl), (NMe_4_)_2_[UCl_6_] (Me = methyl), and (NMe_4_)_2_[UBr_6_] were synthesized following previously published procedures,^90−92^ and their structures confirmed by powder X-ray diffraction (Section S7). The effect of lattice H_2_O molecules on the electronic structure of (NEt_4_)_2_[UF_6_] is discussed in Section S8. Samples were prepared for RIXS measurements as pellets suspended in cellulose. Pellets were sealed behind two layers of Kapton film and mounted in a copper sample holder.

#### Resonant Inelastic X-ray Scattering

Preliminary RIXS measurements were conducted on the MARS beamline at Synchrotron SOLEIL, Saint-Aubin, France.^93−95^ Samples were measured at ambient temperature and pressure. The incident photon energy was selected by a Si(111) double crystal monochromator. The emission energy was selected by a spherically bent Si(220) analyzer. The sample, analyzer crystal, and detector were orientated in a 1 m vertical Rowland geometry. The fluorescence photon intensity was recorded with a KETEK detector.

Further RIXS measurements were conducted at the European Synchrotron Research Facility (ESRF), Grenoble, France. Measurements were performed under vacuum conditions at 10^–6^ bar at base temperature (∼20 K) using the Tender Energy X-ray Spectrometer (TEXS) at the ID26 beamline.^96−98^ The incident photon energy was selected by using the Si(111) reflections of a double silicon crystal monochromator. The incident energy was calibrated by shifting the M_4_ absorption edge energy maximum of [UBr_6_]^2–^ to the theoretical U M_4_ absorption edge value (3726 eV). The U Mα_1,2_ emission photon energies were selected by using one Si(111) analyzer crystal. The U Mβ emission photon energy was selected with one Si(220) analyzer crystal. RIXS planes at the U M_5_ and M_4_ absorption edges were obtained from a series of incident energy scans iterated over an emission energy range centered around the Mα_1,2_ (∼3170 eV) and Mβ (3337 eV) emission lines. The total energy resolution is estimated at a 0.9 and 1.1 eV fwhm at the U M_5_ and M_4_ edges, respectively. The beam size was estimated to be 100 μm by 100 μm. The effects of photon-induced radiolysis for each sample were mitigated by minimizing the scanning time per sample position.

Spurious detector counts due to cosmic rays were removed from affected data sets in postanalysis. The affected data were treated with a linear interpolation of neighboring intensity points across the base of each cosmic ray spike.

### 5.2 Computational

Electronic structure calculations were performed on the [UX_6_]^2–^ complexes (X = F, Cl, and Br) based on single-crystal X-ray diffraction (XRD) crystallographic data. For [UF_6_]^2–^, XRD finds a slight deviation from the *O*_*h*_ point group due to the presence of weakly coordinating H_2_O molecules. Deviation from the *O*_*h*_ point group does not significantly affect the 5*f* ligand field splitting or calculated β values (Section S8). For the calculations reported here, an *O*_*h*_ [UF_6_]^2–^ system utilizing the averaged XRD U–F bond length was used, with H_2_O molecules omitted.

#### Ground State Electronic Structure Calculations

Ground state all-electron (AE) density functional theory (DFT) calculations were performed using the ORCA quantum chemistry software suite.^99^ Scalar relativistic effects were treated using the zeroth order regular approximation (ZORA).^100−102^ The B3LYP functional was used together with the def2-TZVP basis set^103^ for F, Cl, and Br atoms; for U atoms, the segmented all-electron relativistically contracted^89^ SARC-ZORA-TZVPP basis set was used. Auxillary basis sets were utilized by inclusion of the *AutoAux* tag in the input files.

The radial distribution functions of the DFT molecular orbitals of the U(IV) ion and [UX_6_]^2–^ complexes (Figure 5) were calculated with Multiwfn.^104^ The demonstrative radial distribution functions of the U(IV) ion (Figures 12, S2, S3) were obtained at the Hartree–Fock level of theory including relativistic corrections using the Cowan Code Package.^105^

State average (SA) complete active space self-consistent field (CASSCF) calculations were performed at the N-electron-valence perturbation theory (NEVPT2)^106−108^ level using the same combinations of functional and basis set described above. A (2,7) active space was chosen to consist of two electrons in the seven molecular orbitals containing the majority U 5*f* orbital character. Calculations were performed for 21 triplets and 28 singlets. The SA-CASSCF/NEVPT2 results were treated by the *ab initio* ligand field theory (AILFT)^61−63^ method available in ORCA to obtain ground state ligand field parameters.

#### Ligand Field Density Functional Theory Calculations

Average of configuration (AOC) DFT calculations were performed using the Amsterdam Density Functional (ADF) code implemented in the Amsterdam Modeling Suite (AMS, version 2023.104).^67^ The B3LYP functional was used for all of the calculations. Scalar relativistic effects were treated with the zeroth order relativistic approximation (ZORA).^100−102^ All calculations employed Slater type orbital (STO) triple-ζ-plus double polarization all-electron basis sets from the ZORA/TZ2P database.^109^ The active spaces in the AOC calculations were defined as follows. For the ground state, two electrons were equally distributed over the seven 5*f* MOs, each fixed with an electron occupation of 0.286, while for the intermediate and final states, the 5*f* MOs were fractionally populated with three electrons (0.429). The core holes were defined by distributing nine electrons over the core 3*d* orbitals (1.8) and 13 electrons over the core 4*f* orbitals (1.857) in the intermediate and final configurations, respectively. The AOC DFT results were treated by the ligand field density functional theory^68,69^ (LFDFT) implemented in ADF to extract ligand field parameters for each of the initial (5*f*^2^), intermediate (3*d*^9^ 5*f*^3^), and final (4*f*^13^5*f*^3^) RIXS electron configurations.

#### Ligand Field Multiplet Simulations

Multiplet simulations of 3*d*4*f* RIXS planes were carried out using Quanty Version 0.6.^110,111^ The simulations include a Lorentzian broadening of 4.4 and 0.6 eV for the 3*d*^9^ and 4*f*^13^ core-hole lifetimes, in addition to a Gaussian broadening of 0.8 eV. A model Hamiltonian was constructed by using the results of LFDFT calculations to describe the 5*f* ligand field splitting, electron–electron interactions, and spin–orbit coupling in the initial, intermediate, and final electronic configurations of the 3*d*4*f* RIXS process. The influence of each term in the Hamiltonian was systematically investigated to identify the sensitivities of 3*d*4*f* RIXS and to determine the relationship between the observed spectral features and the differences in electronic structure and bonding in going from [UF_6_]^2–^ to [UCl_6_]^2–^ and [UBr_6_]^2–^. The simulations were then fine-tuned with two scaling parameters to obtain the RIXS simulations that best fit the experimental results. Further details can be found in the main text.

## Data Availability

Data Availability Research data files supporting this publication are available from FigShare at https://doi.org/10.6084/m9.figshare.26324782.

## Supporting Information Available

Comments on RXES satellites for U(VI), demonstrative radial distribution function and Slater integral plots, relativistic vs nonrelativistic radial distribution plots, ligand field DFT and multiplet simulation parameters, calculated molecular orbitals from ligand field DFT, full simulated RIXS planes for [UF_6_]^2–^, [UCl_6_]^2–^, and [UBr_6_]^2–^, M_5_-edge HERFD simulations, multiplet simulations showing insensitivities to 5*f* ligand field splitting and spin–orbit coupling, comments on the X-ray crystallographic analysis of the reported compounds and on the presence of water molecules in the crystal structure of [UF_6_]^2–^. (PDF)
