# Correction to “Determination of Uranium Central-Field Covalency with 3*d*4*f* Resonant Inelastic X‑ray Scattering”

**DOI:** 10.1021/jacs.5c14044

**Published:** 2025-10-01

**Authors:** Timothy G. Burrow, Nathan M. Alcock, Myron S. Huzan, Maja A. Dunstan, John A. Seed, Blanka Detlefs, Pieter Glatzel, Myrtille O. J. Y. Hunault, Jesper Bendix, Kasper S. Pedersen, Michael L. Baker

Our work included multiplet simulations of 3*d*4*f* resonant inelastic X-ray scattering (RIXS). Further work has led us to discover that incorrect 4*f* spin–orbit coupling constants were included in the M_4_-edge 3*d*4*f* RIXS simulations. A 4*f* spin–orbit coupling constant of 2.618 eV was erroneously used instead of the ligand field density functional theory (LFDFT) values, as reported in the original manuscript. This led to inaccuracies in the scaling applied to 4*f*–5*f* Slater integrals and, therefore, the nephelauxetic β­(*G*_4*f*5*f*_^0^) factor. Following correction of the 4*f* spin–orbit coupling constant, a less severe scaling to 4*f*–5*f* Slater integrals is required, in closer agreement with LFDFT predicted values. The β­(*F*_5*f*5*f*_^2^) values remain unchanged. The reported sensitivity of 3*d*4*f* RIXS to central field covalency through the quantification of β­(*G*_4*f*5*f*_^0^) and β­(*F*_5*f*5*f*_^2^) is unchanged. The trend in β­(*G*_4*f*5*f*_^0^) is retained, with a larger value for [UF_6_]^2–^ (0.47) than [UCl_6_]^2–^ (0.45) and [UBr_6_]^2–^ (0.44). The correction does not alter or impact the conclusions of the study.

The RIXS simulations shown in Figures 8, 9b, and 10 in the original manuscript are updated in Figures 1, 2, and 3. The corrected RIXS simulations, conducted for the experimental geometry present (analyzer crystal oriented at 84° relative to the incoming X-ray beam), show improved agreement with experiment.

**Figure 1:**
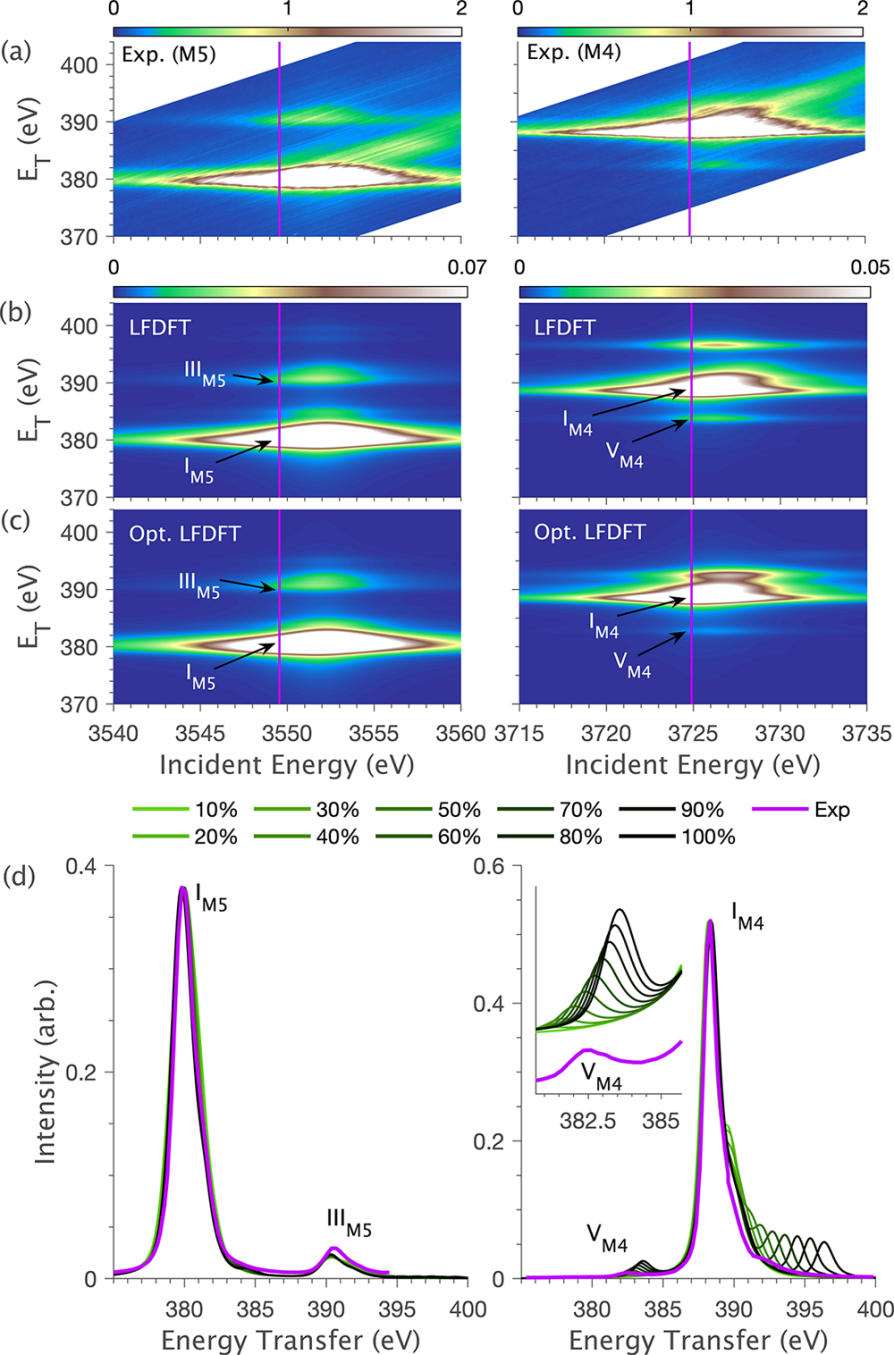
Experimental and simulated RIXS data for [UCl_6_]^2–^ at the M_5_ (left) and M_4_ (right) edges: (a) Experimental RIXS planes; (b) RIXS planes simulated *ab initio* using LFDFT parameters (Tables S3–S8); (c) optimized RIXS planes simulated with a 54% reduction of the LFDFT calculated 4*f*–5*f* Slater integrals to provide the best fit to experiment (optimized values given in Tables S9–S11); (d) RXES cuts showing the stepwise effect of decreasing the magnitude of 4*f*–5*f* Slater integrals.

**Figure 2:**
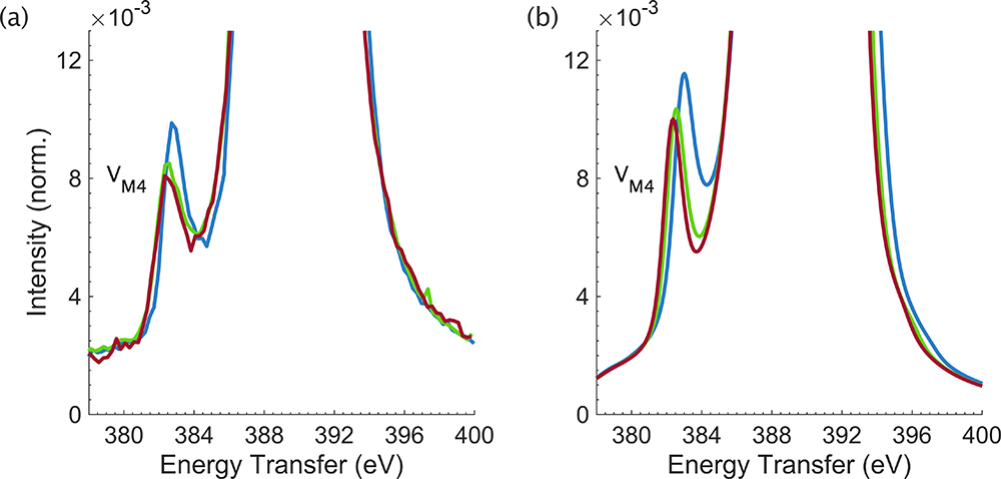
(a) Experimental RXES spectra for [UF_6_]^2–^ (blue), [UCl_6_]^2–^ (green), and [UBr_6_]^2–^ (red). (b) Equivalent RXES cuts through simulated RIXS planes.

**Figure 3:**
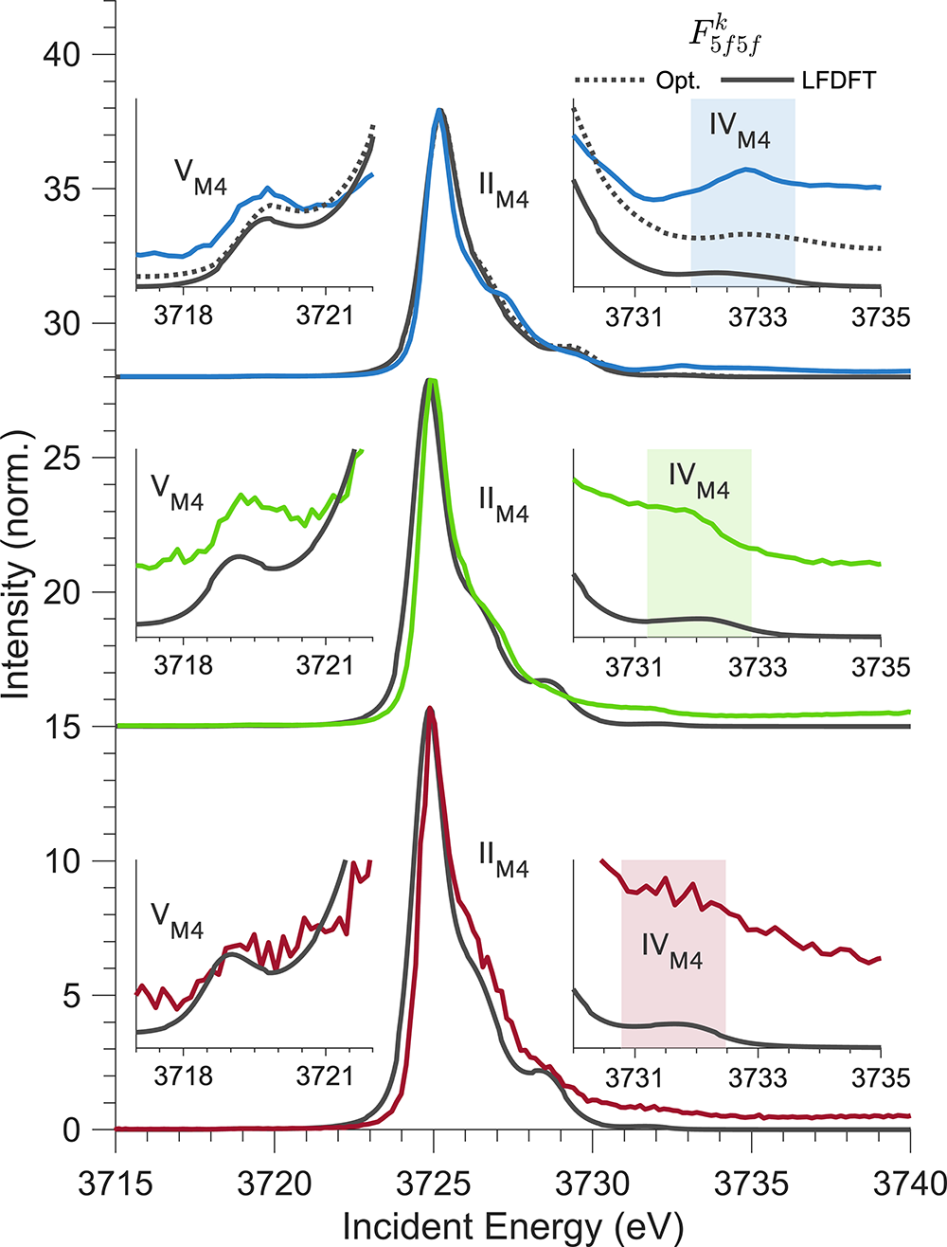
Experimental M_4_-edge HERFD cuts versus the optimized LFDFT simulations for [UF_6_]^2–^ (top), [UCl_6_]^2–^ (middle), and [UBr_6_]^2–^ (bottom). For [UF_6_]^2–^, slight tuning of *F*_5*f*5*f*_^*k*^ is required to accurately reproduce feature IV_M4_, whereas [UCl_6_]^2–^ and [UBr_6_]^2–^ are reproduced using the LFDFT *F*_5*f*5*f*_^*k*^ values. The optimized parameters are given in Tables S9–S11.

Figure 11 and the inlay to Figure 12 in the original manuscript are updated to incorporate the corrected *G*_4*f*5*f*_^0^ values, in Figures 4 and 5. The Supporting Information has also been updated to give the correct calculation parameters (Table S11) and the supplementary simulation (Figures S5–S9).

**Figure 4:**
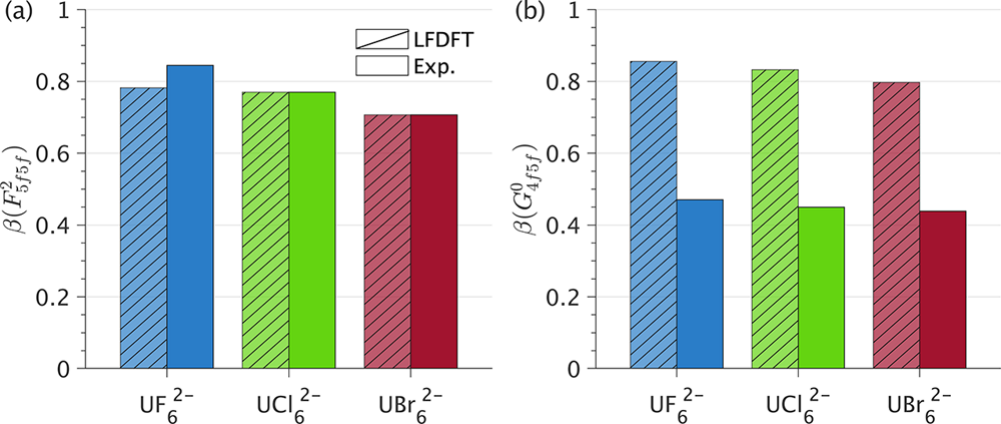
Nephelauxetic reduction parameters (β) for *F*_5*f*5*f*_^2^ (a) and *G*_4*f*5*f*_^0^ (b) determined from LFDFT (hatched bars) and from fitting to 3*d*4*f* RIXS experiment (solid bars).

**Figure 5:**
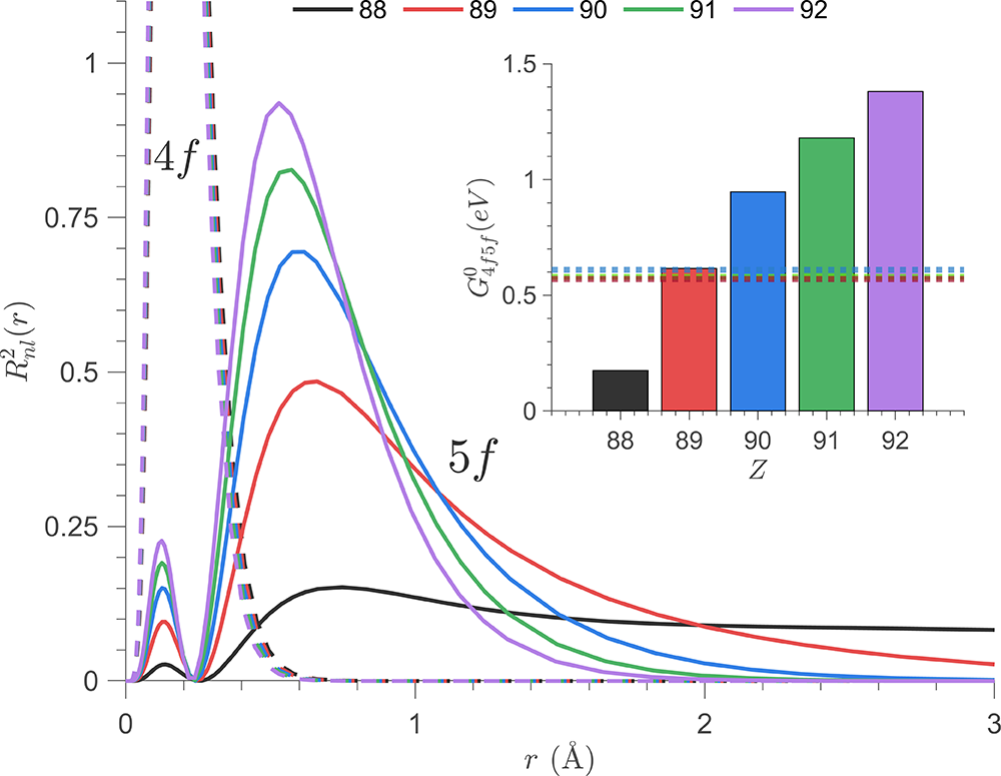
Calculated 4*f* and 5*f* RDFs plotted as a function of nuclear charge (*Z*). (Inlay) Calculated radial integrals for *G*_4*f*5*f*_^0^ plotted as a function of nuclear charge, with experimentally determined values for [UF_6_]^2–^, [UCl_6_]^2–^, and [UBr_6_]^2–^ shown with horizontal dotted lines.

## Supplementary Material



## Notes

Comments on RXES satellite in U­(VI), demonstrative radial distribution function and Slater integral plots, relativistic vs nonrelativistic radial distribution plots, ligand field DFT and multiplet simulation parameters, calculated molecular orbitals from ligand field DFT, full simulated RIXS planes for [UF_6_]^2–^, [UCl_6_]^2–^, and [UBr_6_]^2–^, M_5_-edge HERFD simulations, multiplet simulations showing insensitivities to 5*f* ligand field splitting (corrected) and 5*f* spin–orbit coupling (corrected), comments on the X-ray crystallographic analysis of the reported compounds and on the presence of water molecules in the crystal structure of [UF_6_]^2–^ (PDF)

