# Supplementary material for: Determination of Uranium Central-Field Covalency with 3d4f Resonant Inelastic X-ray Scattering
Source: J Am Chem Soc. 2024 Jul 31;146(32):22570–82. doi: 10.1021/jacs.4c06869 (PMC11328134; doi:10.1021/jacs.4c06869)
Supplement: Supplementary file 1 — ja4c06869_si_001.pdf [file ja4c06869_si_001.pdf]

Supporting Information: Determination of Uranium Central-Field Covalency with $3d4f$ Resonant Inelastic X-ray Scattering

Timothy G. Burrow[&],[†,‡,¶] Nathan M. Alcock[&],[†,‡,¶] Myron S. Huzan,[†,‡,¶] Maja A. Dunstan,[§] John A. Seed,[†,¶] Blanka Detlefs,[‖] Pieter Glatzel,[‖] Myrtille O. J. Y. Hunault,[⊥] Jesper Bendix,[#] Kasper S. Pedersen,[*,§] and Michael L. Baker[*,†,‡,¶]

†*Department of Chemistry, The University of Manchester, Manchester, M13 9PL, U.K.*

‡*The University of Manchester at Harwell, Diamond Light Source, Harwell Campus, OX11 0DE, U.K.*

¶*Centre for Radiochemistry Research, The University of Manchester, Oxford Road, Manchester, M13 9PL, U.K.*

§*Department of Chemistry, Technical University of Denmark, 2800 Kongens Lyngby, Denmark*

‖*European Synchrotron Radiation Facility, 38000 Grenoble, France*

⊥*Synchrotron SOLEIL, L'Orme des Merisiers, 91190 Saint-Auban, France*

#*Department of Chemistry, University of Copenhagen, 1172 Copenhagen, Denmark*

E-mail: kastp@kemi.dtu.dk; michael.baker@manchester.ac.uk

Supporting Information

S1 RXES of $[UO_2Cl_4]^{2-}$ *versus* $[UX_6]^{2-}$

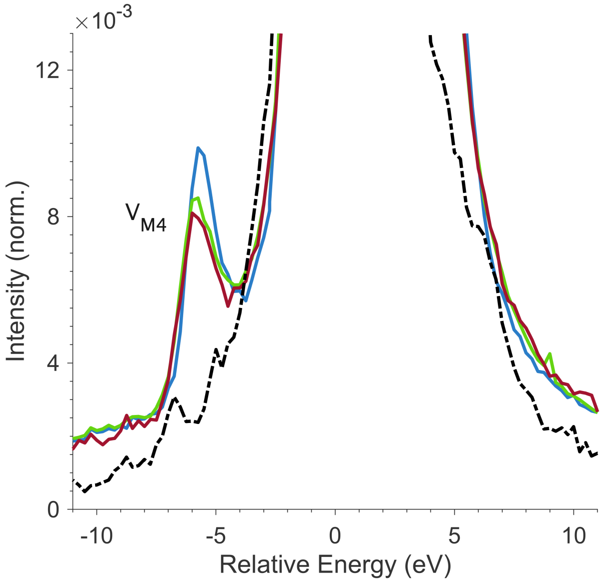

Figure S1: RXES spectra for the U(IV) complexes $[UX_6]^{2-}$ (X = F [blue], Cl [green], Br [red]), compared with the U(VI) complex $[UO_2Cl_4]^{2-}$ (black dotted line). The final state RIXS electron configurations for U(IV) and U(VI) are $4f^{13}5f^3$ and $4f^{13}5f^1$, respectively.

S2 AILFT ligand field parameters

S2.1 Slater integrals & SOC constants

Table S1: AILFT calculated Slater integrals and $5f$ spin-orbit coupling constants for the $5f^2$ ground state, in eV.

	U(IV)	$[UF_6]^{2-}$	$[UCl_6]^{2-}$	$[UBr_6]^{2-}$
F^2_{5f5f}	7.446	7.092	6.656	6.584
F^4_{5f5f}	5.803	5.296	5.275	5.263
F^6_{5f5f}	3.976	3.675	3.607	3.591
ζ_{5f}	0.263	0.244	0.243	0.242

S2.2 Ligand field splittings

Table S2: AILFT calculated ligand field splittings (in eV) for the $5f^2$ ground state.

	$[UF_6]^{2-}$	$[UCl_6]^{2-}$	$[UBr_6]^{2-}$
a_{2u}	0.00	0.00	0.00
t_{2u}	0.29	0.12	0.10
t_{1u}	0.84	0.40	0.33

S3 F2 radial dependence and the influence of relativistic effects

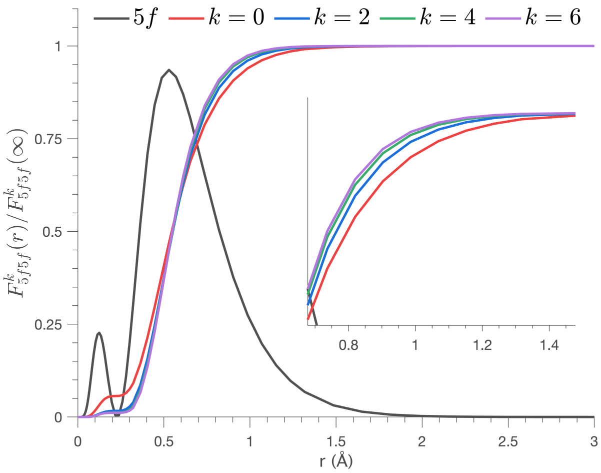

Figure S2: Plots of $F^k_{5f5f}(r)/F^k_{5f5f}(\infty)$ as functions of r compared with $r^2 \cdot R_{5f}{}^2(r)$, calculated for the U(IV) free-ion at the Hartree-Fock theory level using the Cowan Code Package.[1] The inlay highlights how the lower order integrals reach their terminal value at higher radial distances.

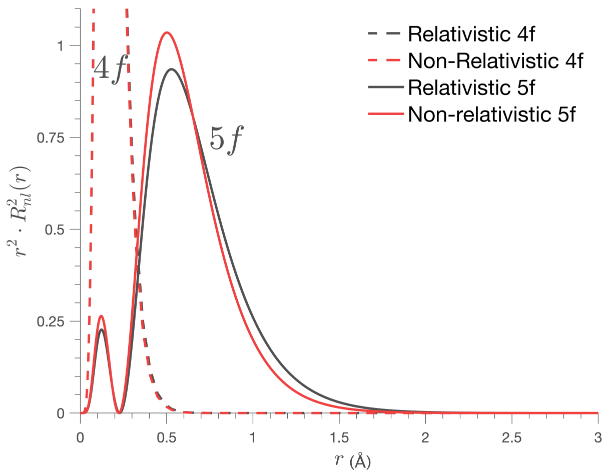

Figure S3: Plots of atomic $5f$ and $4f$ radial distribution functions including and excluding relativistic corrections, calculated for a U(IV) free-ion with a $4f^{13}5f^3$ configuration at the Hartree-Fock theory level using the Cowan Code Package.[1] Values calculated with (and without) relativistic corrections for F^2_{5f5f}: 9.963 eV (10.848 eV); and G^0_{4f5f}: 1.380 eV (1.570 eV) .

S4 Kohn-Sham MOs for $[UX_6]^{2-}$ from AOC calculations

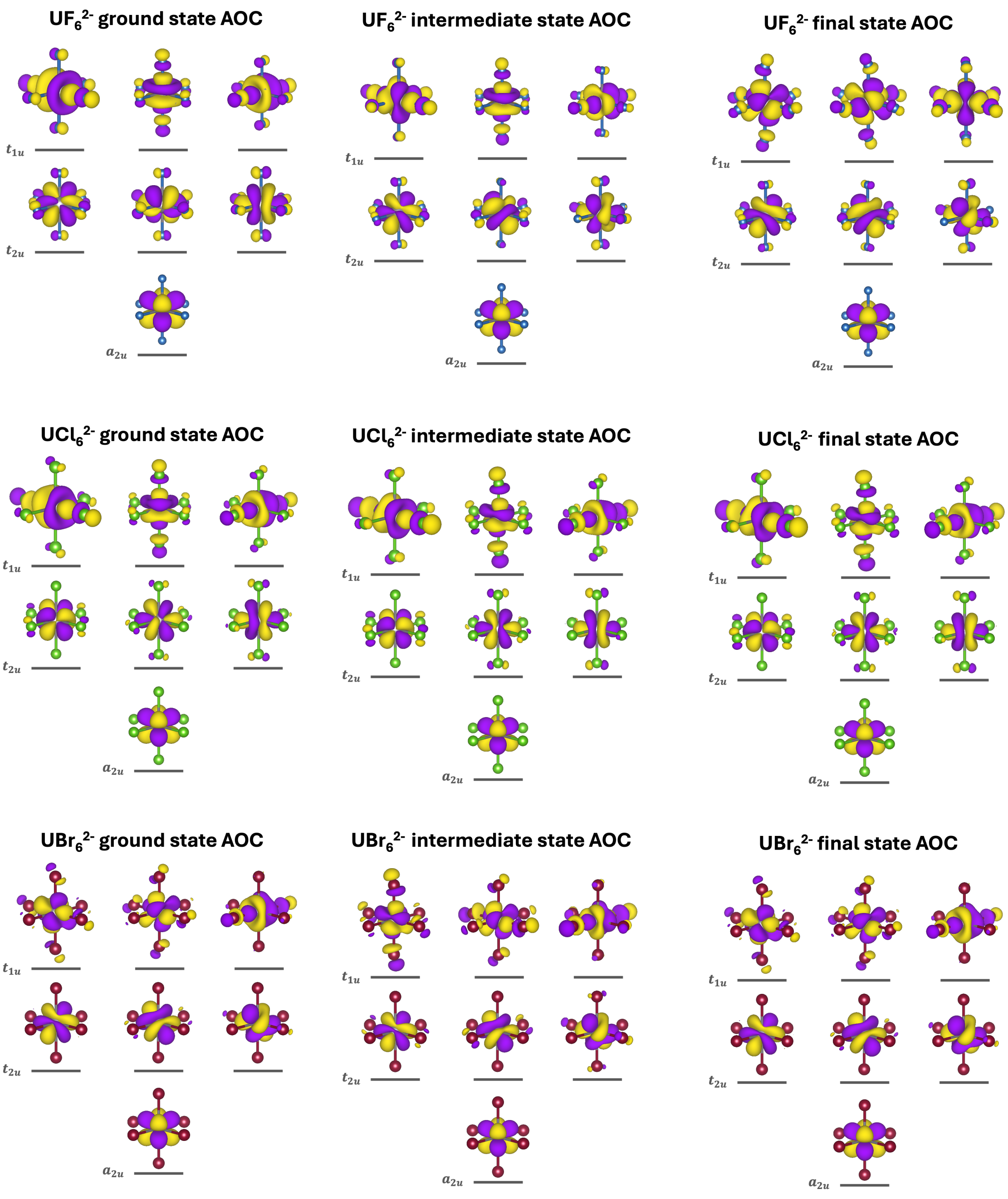

Figure S4: Isosurface plots of the Kohn-Sham molecular orbitals of majority $5f$ character for $[UF_6]^{2-}$, $[UCl_6]^{2-}$, and $[UBr_6]^{2-}$, calculated in the ground ($5f^2$), intermediate ($3d^9 5f^3$), and final ($4f^{13} 5f^3$) state AOC DFT calculations.

S4 LFDFT ligand field parameters

S5.1 Slater integrals & SOC constants

Table S3: LFDFT calculated ground state ($5f^2$) Slater integrals and $5f$ spin-orbit coupling constants, in eV.

	U(IV)	$[UF_6]^{2-}$	$[UCl_6]^{2-}$	$[UBr_6]^{2-}$
F^2_{5f5f}	8.7547	7.0746	7.0353	6.4243
F^4_{5f5f}	5.7099	4.5459	4.5326	4.1476
F^6_{5f5f}	4.1861	3.3111	3.3057	3.0273
ζ_{5f}	0.2495	0.2039	0.2103	0.2016

Table S4: LFDFT calculated intermediate state ($3d^95f^3$) Slater integrals and spin-orbit coupling constants, in eV.

	U(IV)	$[UF_6]^{2-}$	$[UCl_6]^{2-}$	$[UBr_6]^{2-}$
F^2_{3d5f}	2.3988	2.0597	2.0024	1.9183
F^4_{3d5f}	1.1196	0.9589	0.9309	0.8917
G^1_{3d5f}	1.8627	1.5954	1.5486	1.4835
G^3_{3d5f}	1.1316	0.9693	0.9407	0.9012
G^5_{3d5f}	0.7932	0.6794	0.6594	0.6317
F^2_{5f5f}	8.9533	7.0347	6.9033	6.3430
F^4_{5f5f}	5.8604	4.5867	4.4734	4.1148
F^6_{5f5f}	4.3053	3.3632	3.2719	3.0108
ζ_{3d}	70.6995	70.7003	70.7007	70.701
ζ_{5f}	0.2810	0.2408	0.2337	0.2239

Table S5: LFDFT calculated final state ($4f^{13}5f^3$) Slater integrals and spin-orbit coupling constants, in eV.

	U(IV)	$[UF_6]^{2-}$	$[UCl_6]^{2-}$	$[UBr_6]^{2-}$
F^2_{4f5f}	4.8684	4.1937	4.0965	3.9230
F^4_{4f5f}	2.1134	1.8086	1.7611	1.6859
F^6_{4f5f}	1.3107	1.1195	1.0895	1.0427
G^0_{4f5f}	1.2994	1.1117	1.0816	1.0359
G^2_{4f5f}	1.6254	1.3883	1.3504	1.2924
G^4_{4f5f}	1.2703	1.0844	1.0548	1.0095
F^2_{5f5f}	8.9440	6.9921	6.8863	6.3213
F^4_{5f5f}	5.8566	4.5609	4.4637	4.1021
F^6_{5f5f}	4.3035	3.3452	3.2654	3.0021
ζ_{4f}	3.0455	3.0447	3.0447	3.0445
ζ_{5f}	0.2776	0.2372	0.2306	0.2208

S5.2 Ligand field splittings

Table S6: LFDFT calculated ligand field splittings for the $5f^2$ ground state, in eV.

	$[UF_6]^{2-}$	$[UCl_6]^{2-}$	$[UBr_6]^{2-}$
a_{2u}	0.00	0.00	0.00
t_{2u}	0.56	0.13	0.10
t_{1u}	1.35	0.35	0.26

Table S7: LFDFT calculated ligand field splittings for the $3d^95f^3$ intermediate state, in eV.

	$[UF_6]^{2-}$	$[UCl_6]^{2-}$	$[UBr_6]^{2-}$
a_{2u}	0.00	0.00	0.00
t_{2u}	0.31	0.13	0.10
t_{1u}	0.82	0.35	0.27

Table S8: LFDFT calculated ligand field splittings for the $4f^{13}5f^3$ final state, in eV.

	$[UF_6]^{2-}$	$[UCl_6]^{2-}$	$[UBr_6]^{2-}$
a_{2u}	0.00	0.00	0.00
t_{2u}	0.31	0.13	0.10
t_{1u}	0.83	0.36	0.27

S6 LFDFT RIXS calculations

S6.1 LFDFT RIXS planes

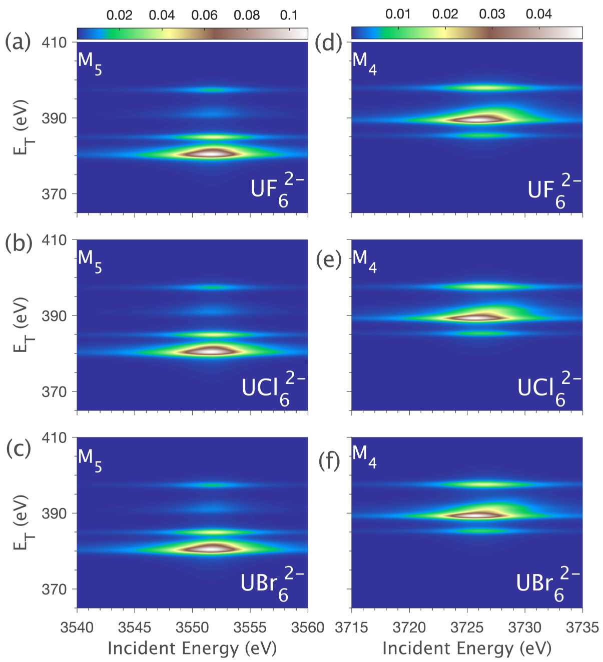

Figure S5: $3d4f$ RIXS planes calculated using LFDFT deduced values at the M_5 (a-c) and M_4 (d-f) edges for $[UX_6]^{2-}$.

S6.2 Optimised LFDFT RIXS planes

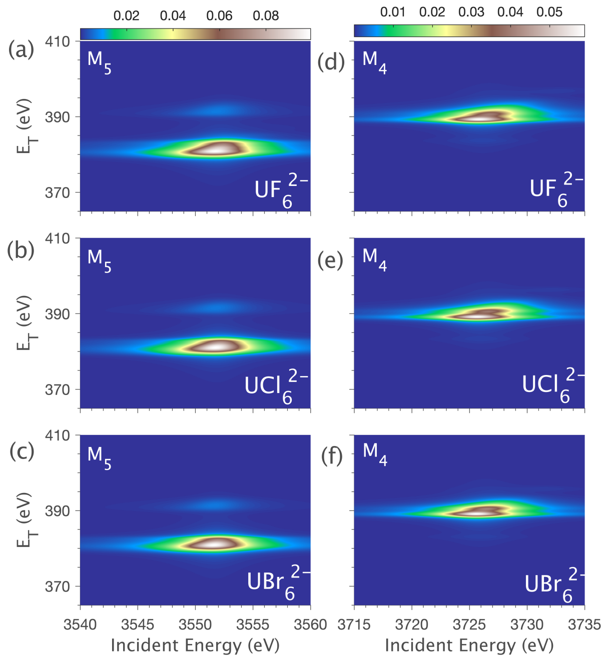

Figure S6: Optimised $3d4f$ RIXS calculations at the M_5 (a-c) and M_4 (d-f) edges for $[UX_6]^{2-}$, calculated using the optimised parameter sets in Tables S9-S11.

Table S9: Optimised ground state ($5f^2$) Slater integrals and $5f$ spin-orbit coupling constants, in eV.

	$[UF_6]^{2-}$	$[UCl_6]^{2-}$	$[UBr_6]^{2-}$
F^2_{5f5f}	7.6406	7.0353	6.4243
F^4_{5f5f}	4.9096	4.5326	4.1476
F^6_{5f5f}	3.5760	3.3057	3.0273
ζ_{5f}	0.2039	0.2103	0.2016

Table S10: Optimised intermediate state ($3d^9 5f^3$) Slater integrals and spin-orbit coupling constants, in eV.

	$[UF_6]^{2-}$	$[UCl_6]^{2-}$	$[UBr_6]^{2-}$
F^2_{3d5f}	2.0597	2.0024	1.9183
F^4_{3d5f}	0.9589	0.9309	0.8917
G^1_{3d5f}	1.5954	1.5486	1.4835
G^3_{3d5f}	0.9693	0.9407	0.9012
G^5_{3d5f}	0.6794	0.6594	0.6317
F^2_{5f5f}	7.5975	6.9033	6.3430
F^4_{5f5f}	4.9536	4.4734	4.1148
F^6_{5f5f}	3.6323	3.2719	3.0108
ζ_{3d}	70.7003	70.7007	70.701
ζ_{5f}	0.2408	0.2337	0.2239

Table S11: Optimised final state ($4f^{13} 5f^3$) Slater integrals and spin-orbit coupling constants, in eV.

	$[UF_6]^{2-}$	$[UCl_6]^{2-}$	$[UBr_6]^{2-}$
F^2_{4f5f}	0.9629	0.8357	0.8356
F^4_{4f5f}	0.4153	0.3593	0.3591
F^6_{4f5f}	0.2570	0.2223	0.2221
G^0_{4f5f}	0.2552	0.2206	0.2206
G^2_{4f5f}	0.3188	0.2755	0.2753
G^4_{4f5f}	0.2490	0.2152	0.2150
F^2_{5f5f}	7.5515	6.8863	6.3213
F^4_{5f5f}	4.9258	4.4637	4.1021
F^6_{5f5f}	3.6128	3.2654	3.0021
ζ_{4f}	3.0447	3.0447	3.0445
ζ_{5f}	0.2372	0.2306	0.2208

S6.3 Sensitivity of RIXS simulations to LFS and SOC

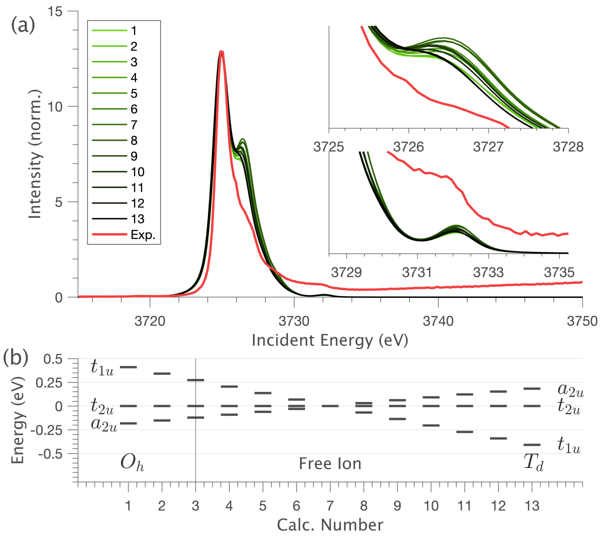

Figure S7: (a) Multiplet calculations assessing the $5f$ ligand field dependence upon the M_4-edge HERFD. (b) Scaling of the $5f$ ligand field is relative to the calculated LFDFT values for $[UCl_6]^{2-}$ (calculation 3, vertical grey line), surveying from O_h to T_d ordering with all other parameters set to the optimised $[UCl_6]^{2-}$ values (Tables S9-S11).

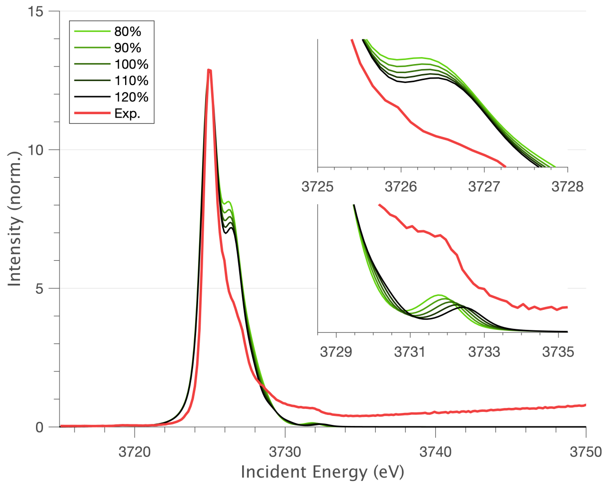

Figure S8: Multiplet calculations assessing the ζ_{5f} dependence upon the M_4-edge HERFD. Scaling of ζ_{5f} is relative to the calculated LFDFT value for $[UCl_6]^{2-}$ with all other parameters set to the optimised $[UCl_6]^{2-}$ values (Tables S9-S11).

S6.4 M_5-edge HERFD simulations

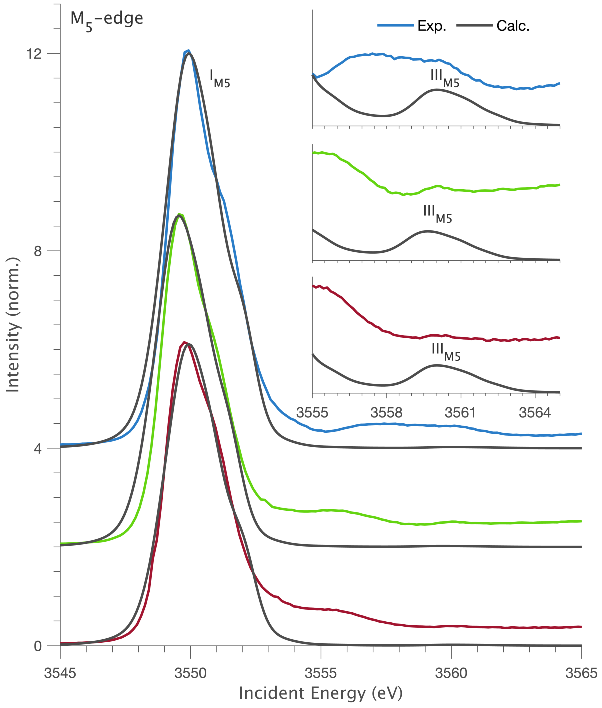

Figure S9: Experimental M_5-edge HERFD cuts (colored lines) *versus* simulations (black lines) using optimised $4f$-$5f$ Slater integrals for $[UF_6]^{2-}$ (*top*), $[UCl_6]^{2-}$ (*middle*), and $[UBr_6]^{2-}$ (*bottom*). The experimental spectra are reproduced without further tuning of the LFDFT calculated F^k_{5f5f} values (Tables S3-S5).

S7 X-ray diffraction and crystallography

Single crystals of the new solvatomorph $(NEt_4)_2[UF_6]\cdot 7H_2O$ were grown by slow diffusion of acetone into the propylene carbonate reaction solution over one week. Single crystals of $(NMe_4)_2[UBr_6]$ were grown by slow diffusion of degassed diethyl ether into a degassed aqueous solution of the compound over 24 hours. Single crystal X-ray diffraction data were collected on an Oxford Diffraction Supernova single crystal diffractometer, with an Atlas CCD detector. Crystals were taken from solution directly into polybutene oil in a glovebox and mounted on a nylon loop for measurement. Data were collected at 120 K using Cu Kα radiation ($\lambda = 1.54184$) Å). Single crystal X-ray diffraction data were reduced using CrysAlisPro[2] and corrected using a numerical absorption correction based on Gaussian integration

over a multi-faceted crystal model. The structure was solved with the SHELXS[3] structure solution program using direct methods and refined with the SHELXL[4] refinement package using least squares minimization on all data, in Olex2.[5] The compound $(NMe_4)_2[UBr_6]$ is isomorphous to the previously reported $(NMe_4)_2[UCl_6]$.[6]

S8 Effect of H_2O molecules on electronic structure of $[UF_6]^{2-}$

The crystal structure analysis of $[UF_6]^{2-}$ reveals the presence of H-bonds between the F^- ions and molecules of H_2O present in the structure. Each unit of $[UF_6]^{2-}$ exhibits these weak interactions for five of the bonding F^- ligands. The presence of these H bonds has a minimal effect on the U-F bond lengths, causing variations within ±0.015 Å of the average, 2.173 Å. The sixth F atom is also within ±0.015 Å of the average bond length, with a U-F distance of 2.163 Å.

To assess the effect of the presence of the H_2O molecules on the electronic structure of $[UF_6]^{2-}$, ground state *ab initio* calculations were performed on three different systems: **1** the crystal structure of $[UF_6]^{2-}$ without the H_2O molecules, **2** the crystal structure of $[UF_6]^{2-}$ including the H_2O molecules, and **3** a perfectly O_h $[UF_6]^{2-}$ system, using the average bond length (2.173 Å) and adjusted 90° inter-bond F-U-F angles. Table S12 shows the AILFT calculated parameters.

Table S12: AILFT derived ligand field splittings (in eV) calculated at the SA-CASSCF/NEVPT2 level of theory. For each of the three systems, the Slater integral F^2_{5f5f} is given (in eV), and is also expressed as a fraction of the the value calculated for a free U(IV) ion, β.

**System**	**1**	**2**	**3**
a_{2u}	0	0	0
t_{2u}	0.29	0.28	0.29
t_{1u}	0.84	0.82	0.84
${F^2}_{5f5f}$	7.10	7.09	7.09
β	0.95	0.95	0.95

There are no differences in the calculated β values with the inclusion of the H-bonding

molecules of H_2O. There is a minor change in the ligand field splitting for system **2**: the t_{1u} is reduced by 0.2 eV and the t_{2u} by 0.1 eV. This is intuitive, as the weak H-F hydrogen bonds slightly reduce the electron density on F, resulting in a weaker ligand field splitting of the U $5f$ orbitals. However, due to the insensitivity of the RIXS simulations to the $5f$ ligand field splittings (Section S6.3), and the overall negligible differences between **1**, **2** and **3**, the simple O_h model **3** was used in subsequent calculations, as reported in the main text.

Author Contributions

[&] T.G.B. and N.M.A. contributed equally to this work as co-first authors.

References

(1) Cowan, R. D. *The Theory of Atomic Structure and Spectra*; University of California Press, 1981; p 731.

(2) CrysAlisPRO, Oxford Diffraction /Agilent Technologies UK Ltd, Yarnton, England. 2014.

(3) Sheldrick, G. M. A short history of SHELX. *Acta Crystallographica Section A: Foundations of Crystallography* **2008**, *64*, 112–122.

(4) Sheldrick, G. M. Crystal structure refinement with SHELXL. *Acta Crystallographica Section C: Structural Chemistry* **2015**, *71*, 3–8.

(5) Dolomanov, O. V.; Bourhis, L. J.; Gildea, R. J.; Howard, J. A. K.; Puschmann, H. *OLEX2*: a complete structure solution, refinement and analysis program. *Journal of Applied Crystallography* **2009**, *42*, 339–341.

(6) Autillo, M.; Wilson, R. E. Phase Transitions in Tetramethylammonium Hexachlorometalate Compounds $(TMA)_2MCl_6$ (M = U, Np, Pt, Sn, Hf, Zr). *European Journal of Inorganic Chemistry* **2017**, 4834–4839.
